# Effect of the axial load on the dynamic response of the wrapped CFRP reinforced concrete column under the asymmetrical lateral impact load

**DOI:** 10.1371/journal.pone.0284238

**Published:** 2023-06-02

**Authors:** Khalil AL-Bukhaiti, Liu Yanhui, Zhao Shichun, Hussein Abas, Han Daguang, Xu Nan, Yang Lang, Yan Xing Yu

**Affiliations:** 1 School of Civil Engineering, Southwest Jiaotong University, Chengdu, Sichuan, China; 2 Faculty of Civil Engineering, Southeast University, Nanjing, China; National University of Sciences and Technology, PAKISTAN

## Abstract

This study investigated the impact of axial load on the dynamic response of reinforced concrete (RC) members to asymmetrical lateral impact loads. A series of asymmetrical-span impact tests were conducted on circular and square RC members with and without Carbon Fiber Reinforced Polymers (CFRP) while varying the axial compression ratios. The impact process was simulated using ABAQUS software, and the time history curves of deflection and impact were measured. The study found that specific impact loads caused bending and shearing failures. The axial compression ratio ranged from 0.05 to 0.13 when the impact curve reached its maximum deflection before the component’s impact resistance decreased. Analysis of the impact point and inclined crack location revealed that axial load affects the maximum local concrete. The speed of inclined crack penetration and inclined cracks take longer to form, with weaker resistance to damage to local concrete when the axial compression ratio is between 0.05 and 0.13. When the axial compression ratio is greater than 0.13, inclined cracks form sooner with more brittle and severe damage to the impact point’s concrete. The study also identified key parameters affecting the dynamic response of RC members, including impact height, CFRP layer thickness, axial force, and impact location. Thicker CFRP layers in RC can improve impact resistance, especially when the impact location is farther from the center. However, there is a limit to the impact of axial force on this resistance.

## 1. Introduction

Impact force studies focus on understanding the forces involved in collisions and impacts between two objects. The novelty of these studies lies in their ability to provide insight into the mechanisms and effects of such impacts, which can be used to improve the design of structures and materials to make them more resistant to damage from impact forces. This can have applications in various fields, including engineering, physics, and materials science. Reinforced concrete structures often need to be designed to withstand impact loads, which can arise from the collision of relatively rigid heavy objects at low velocities, such as falling rocks in mountainous regions and dropped loads in industrial and warehouse settings due to accidents. It is crucial to evaluate the structural integrity of RC structures under impact loading to develop a performance-based design approach that meets current impact loading specifications, such as those outlined by the American Association of State Highway and Transportation Officials [1] and the UK Atomic Energy Authority [2]. Impact loading is a highly severe condition characterized by applying intense force over a short period. The behavior of a structural component under impact loading may involve two response phases, the local response resulting from the stress wave at the loading point during the initial moments of impact, and the overall response, including the free vibration effect stemming from the elastic-plastic deformation that occurs over a longer period throughout the entire structural member after the impact [3, 4]. The loading rate effect and the dynamic behavior of the structural component heavily influence the overall response. Reinforced concrete (RC) columns have good mechanical properties and are often used in structures like bridges, high-speed rail stations, and underground parking lots for their load-bearing capabilities. During use, ships, vehicles, or high-speed trains may hit reinforced concrete columns. It may result in huge economic losses and casualties if the components are not impact-resistant. In light of this, it is of great practical importance to study the dynamic response of reinforced concrete columns subjected to impact loads. In recent years, CFST and reinforced concrete members have been subjected to numerous studies on impact resistance, and progress has been made in some areas. Researchers developed new impact testing methods, investigated the effects of impact force on different materials and structures, and developed materials and designs that can withstand impact forces [4–12]. Pham et al. [10] studied how the axial force, or the force exerted along the main axis of the concrete structure, affected the behavior and performance of reinforced concrete when subjected to impact loading. His research found that the axial force significantly influenced the concrete’s ability to withstand impact loading. Depending on the specific conditions and loading scenarios, it could either enhance or degrade the material’s performance. Overall, his research aimed to provide insights into the mechanisms behind the behavior of reinforced concrete under impact loading and to help inform design and construction practices for structures that may be subjected to such loads. Structural engineering researchers have widely studied axial force’s effect on reinforced concrete under impact loading. It is well known that reinforced concrete structures are highly resistant to static loads, but their behavior under dynamic loads, such as those caused by earthquakes or impacts, is less predictable. Swesi et al. [13] focused on the effect of axial force on the response of reinforced concrete columns under impact loading. They conducted experiments on concrete columns with various levels of axial force and found that the axial force had a significant effect on the response of the columns. In general, they found that increasing the axial force led to increased peak force and energy absorbed by the columns and reduced displacement and lateral deformations. Li et al. also examined the dynamic response of reinforced concrete beams under impact loading [14]. They found that the axial force significantly affected the peak force and energy absorbed by the beams, as well as the maximum displacement and lateral deformations. They also found that axial force’s effect on the beams’ dynamic response was more pronounced at higher impact velocities. In addition to these experimental studies, several numerical simulations have also been conducted to investigate the effect of axial force on reinforced concrete under impact loading. One such study by Jin et al. [15] used finite element analysis to examine the effect of axial force on the dynamic response of reinforced concrete columns under impact loading. They found that the axial force significantly affected the peak force and energy absorbed by the columns, as well as the maximum displacement and lateral deformations. Overall, when studying the dynamic response of RC components under the impact, it is clear that axial compression (hereinafter referred to as axial force) on reinforced concrete under impact loading is significant and cannot be ignored [16, 17]. How the axial force is effectively applied to the member is one of the issues to be considered in the research. Structural engineers need to consider the effect of axial force when designing reinforced concrete structures to ensure their safety and reliability under dynamic loads. Based on the literature studies, it is of great significance to study the effect of axial force on the dynamic response of RC components under impact. This paper takes the derailed train impacting the RC column with/out CFRP layers of the station building as the research object, carries out the asymmetrical span impact test, and studies the RC member’s dynamic response and failure mechanism under the impact and axial force. As a result, this study takes the square and circular RC members as the object. It uses the high-performance drop weight test system of the Taiyuan University of Technology to carry out the lateral impact test. In the test, an initial axial force is applied to the test member through the designed self-balancing and stable axial compression system and sliding support system. The boundary conditions are free in the axial and fixed in other directions. An investigation of the effect of axial forces on the deflection time history, failure mode, and impact curves of a member is conducted using the impact force measured by drop weight tests. After validating the test output via ABAQUS 2020, different parametric studies were established, such as the maximum principal stress of concrete, shear crack penetration time, and strengthening the RC members with CFRP layers (Effect of CFRP layers thickness, impact position, bending moment, energy absorbed, and the stress of components) are analyzed. In conclusion, the novelty of this study will contribute to a better understanding of the effect of axial compression on the dynamic response of CFRP-RC components to lateral impact, including bending moment, energy change, and stress development. Also, understanding the effect of the axial compression ratio plays a critical role in the dynamic response of reinforced concrete members to lateral impact load and that the parameters explored in this study can be leveraged to improve impact resistance.

## 2. Experimental program

**Fig 1:**
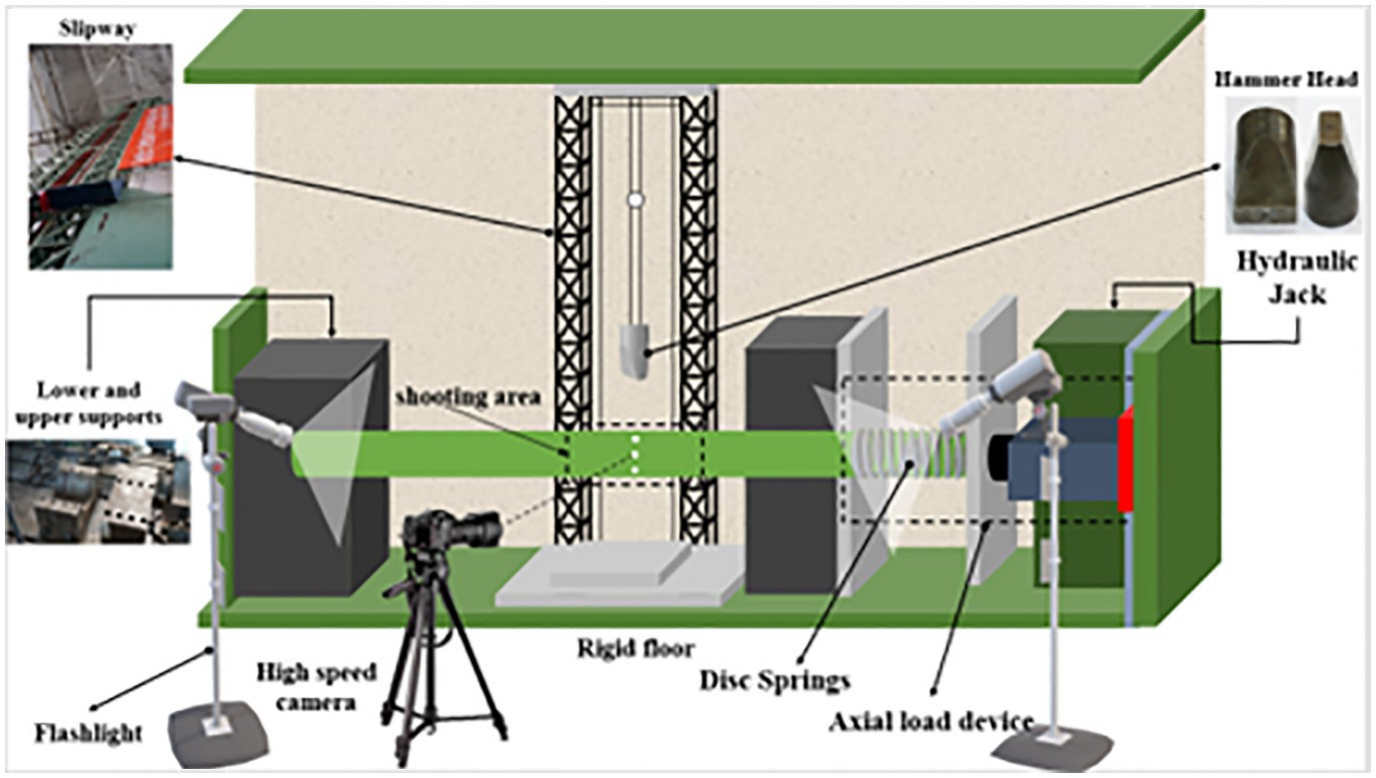
3-D schematic diagram of DHR9401 drop weight impact test equipment with the axial load device.

The impact test was conducted on a DHR9401 drop-weight impact testing machine Fig 1 from the Taiyuan University of Technology. The test’s main purpose is to study the dynamic response of RC members under impact and provide data for finite element analysis. The test device is mainly composed of fixed left and roller right supports, an impact body, a drop weight guide rail, and an axial force loading device; the effective height of the test machine is 12.6m, and the maximum impact speed can reach 15.7m/s.

**Fig 2:**
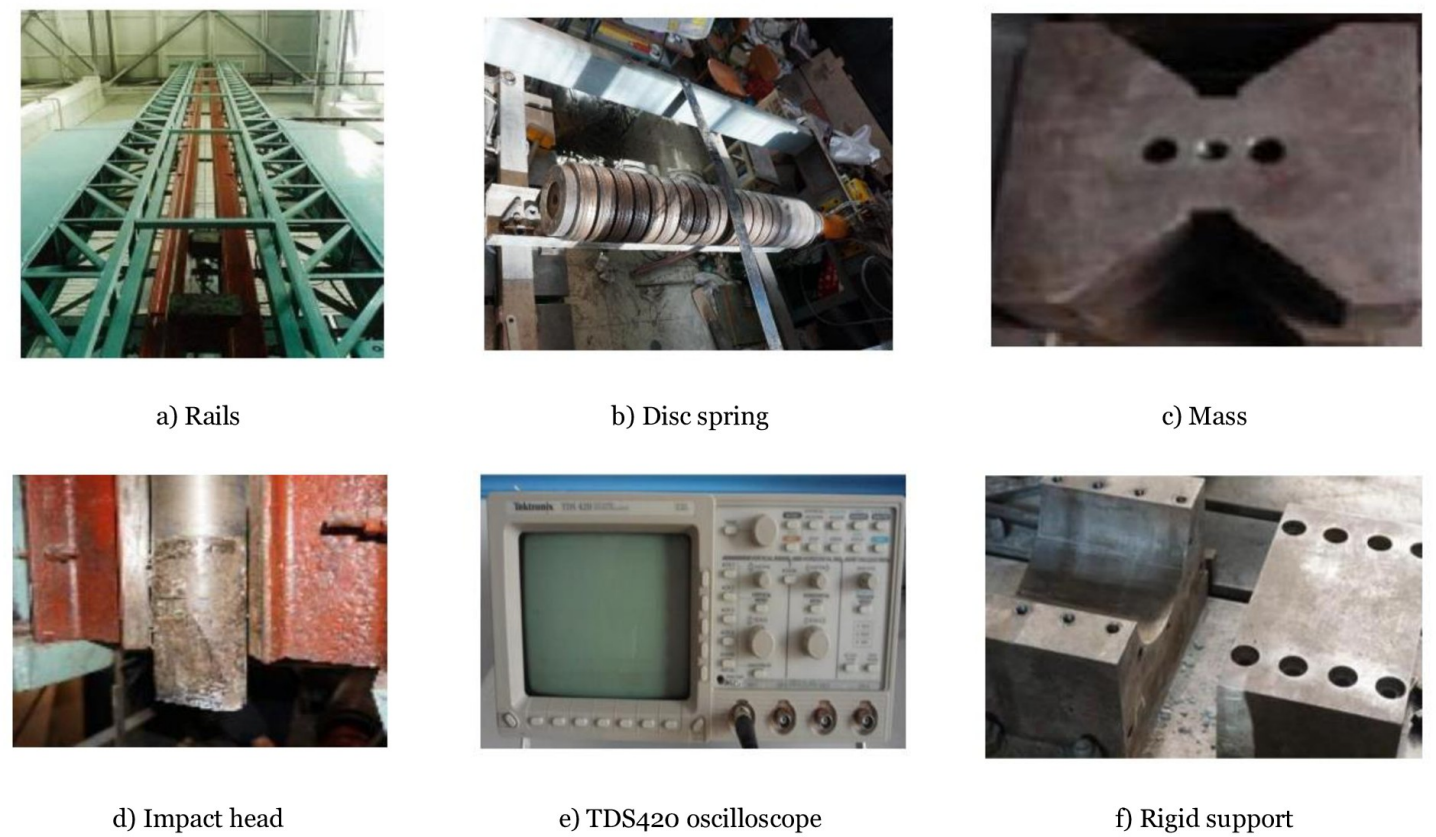
Test equipment diagram. a) Rails, b) Disc spring, c) Mass, d) Impact head, e) TDS420 oscilloscope, f) Rigid support.

The impact body consists of an impact head and several mass blocks Fig 2. The impact head’s contact surface is rectangular, 80mm long, and 30mm wide. It is made of chrome with a Mohs hardness of 8.5 (with negligible deformation), the mass of the impact body is set to 230kg, and a mechanical sensor is installed between the impact head and the mass to record the impact force time history curve. The axial force loading device comprises a hydraulic compressor, a spring group, and a mechanical sensor. A 50t hydraulic compressor controlled by displacement is used to apply an axial force to the specimens. To prevent the axial force from disappearing or reducing instantaneously during the impact process, the deformation stored by the spring group can keep the axial force unchanged during the impact process. The mechanical sensor is fixed between the spring and the jack to measure the value of the axial force, and the test apparatuses are shown in Fig 2.

The test data acquisition system consists of a force sensor installed on the impact body, the hydraulic press, and a high-speed camera set in front of the test bench. The impact force time-history curve is recorded by the force sensor set at the impact head position, using the TDS420 oscilloscope produced by American Tektronix to store the data, and the recording frequency is 1MHZ. A high-speed camera captures the impact image process with a frame rate of 2500FPS. After post-processing the captured image, a member deformation time-history curve can be obtained every 0.4ms.

### 2.1 Test specimens

The derailment train hitting the high-speed railway station building has a relatively small shear span of the components in the building structure. The height of the RC column is mostly 10-12m, and the diameter is about 1.2m. The impact point of the derailed train is about 1.8m above the column bottom [18]. CRH2 trains have high speed and large impact energy, and the resistance of components is relatively small. Considering the equipment conditions during the test, the scale ratio of the designed RC test members is 1:10 square, and circular reinforced concrete members are designed. The member information is shown in Table 1.

**Table 1:** Shows the member’s information details.

No.*	Longitudinal reinforcement ratio/%	Stirrup’s ratio/%	Axial force/kN	Basic conditions
**FH2**	2.01%(4∅8)	0.62 (∅4@50)	0	Impact body mass 230kg, impact height 2m, impact energy 4513J
**FH5**			200	
**YH2**	1.67%(6∅6)	1.26% (∅4@50)	0	
**YH5**			200	

*Note: "F" means square section, "Y" means circular section, and "H" means reinforced concrete.

**Fig 3:**
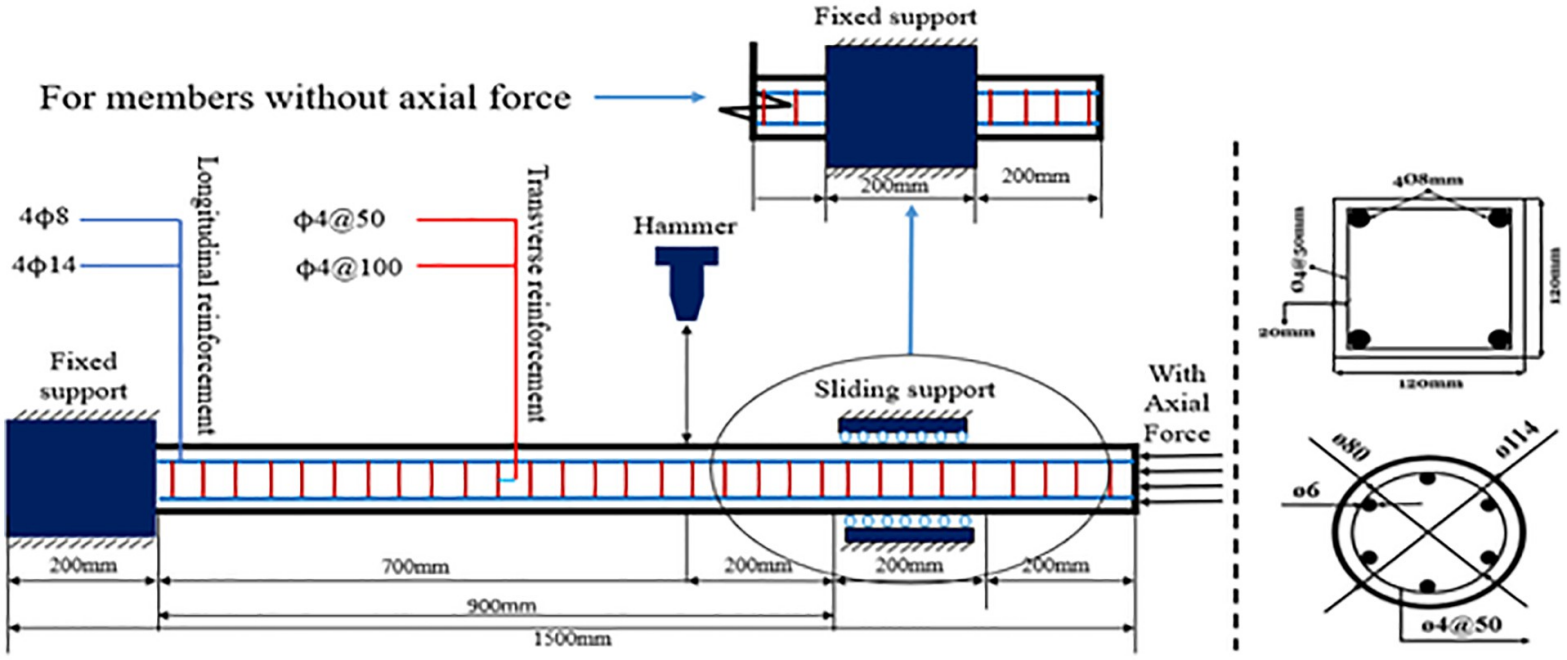
Member design drawing (unit: mm).

As shown in Fig 3, the design length of the test member is 1500mm, the effective support length is 900mm, the left side is fixed, and the right side can slide axially; the test is an asymmetrical span impact force, and the impact point is 200mm away from the right support. The section size of the circular members is 115mm and 120*120mm for square members. The thickness of the concrete cover layer is 20mm, and the reinforcement details are shown in Fig 3.

### 2.2 Material characteristics

According to "Standards for Experimental Methods of Mechanical Properties of Ordinary Concrete" (GB/T 50081–2002), the average 28-day cube compressive strength of concrete was measured to be 54.97MPa for circular specimens and 43.5MPa for square specimens, the material experimental test figures as mentioned in the author’s previous works [18, 19]. The tensile test of the steel bar was carried out according to the "Test method for tensile test of metallic materials at room temperature" (GB/T 228–2010). The mechanical properties of the steel bar are shown in [18–21].

## 3. Experimental results

### 3.1 Effect of axial force on the failure mode

**Fig 4:**
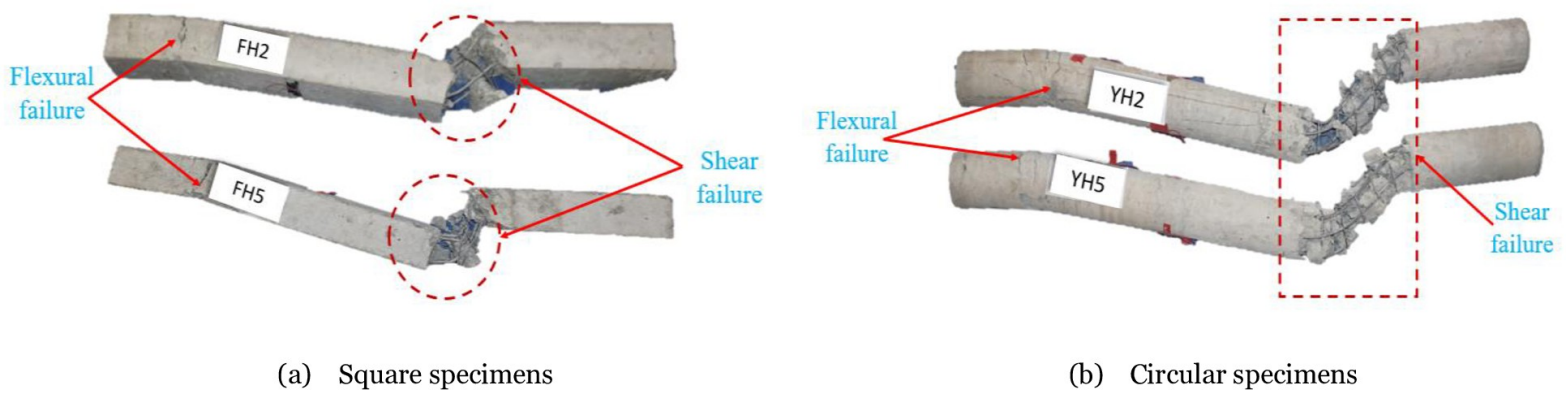
Failure modes after the end of the impact scenario for all specimens. (a) Square specimens, (b) Circular specimens.

A high-speed camera captures the failure modes of the members, and the images of the failure process of the three members during the impact process are intercepted for analysis. For square members, the final failure mode of the member is shown in Fig 4a. The concrete at the impact point of the FH2 member has a small area of spalling, and the stirrup is broken. A significant portion of the concrete in the impact zone of the axially loaded FH5 member has undergone significant peeling, accompanied by extensive fracture of the stirrups and longitudinal reinforcements. In summary, the damage degree of the RC members is more serious after the axial load is applied, the concrete at the impact point is damaged and spalled in a large area, and the number of fractures of the longitudinal reinforcement and stirrup increases. The resistance of the FH2 and FH5 members is not enough to withstand the impact energy applied in the test, and the members are severely damaged, and the damage occurs quickly. It is difficult to collect data on the impact of the axial force on the dynamic response of the RC members completely and effectively. For circular members, it seems all members exhibited a brittle shear failure and massive concrete cracking with buckling of the steel reinforcement bars in the diagonal crack region. For the sake of observation, in Fig 4b, the arrows are the occurrence of flexural cracks in the left support, and the inside of the rectangular dotted line is the brittle shear failure. The damaged concrete has lost its bearing capacity at the impact point. Only the steel bars in this area can continue to afford. The deformation generated by the impact exceeded the steel’s full strain capacity, indicating that the specimens have lost the overall force mechanism. Fig 4b reveals that, due to the asymmetrical impact force location, shear failure at the impact point with vertical cracks at the upper end of the left support was increased, as observed in member YH2. This finding suggests that members close to the left support area are primarily subjected to bending damage. This observation provides insight into the underlying reasons for the failure of RC members. For member YH5, the failure mode indicates catastrophic shear failure and massive local failure due to axial load, resulting in the buckling of longitudinal steel near the impact zone. The failure areas along the longitudinal direction of the members are relatively limited. When the hammer hits the RC members, all the regions near the impact zone fail, indicating no significant damage, except in the left support region, for cross-sections located somewhat away from the impact point. These findings are consistent with the available experimental works and previous literature, which also arrived at the same conclusion [7, 22, 23].

### 3.2 Effect of the axial force on the impact time history

In the case of applying axial force with square members, the FH5 impact force curve changes significantly. FH2 and FH5 components have the same impact energy, but the peak impact force is reduced by 42%. In the section on failure modes above, we found that at the beginning of the impact process of the FH5 member, the local concrete at the impact point was greatly damaged and spalled, which made the contact stiffness at this position smaller, resulting in a decrease in the peak value of FH5. After the peak period, the FH5 impact force curve fluctuates greatly, and there is no plateau with a long duration. The curve oscillates violently between 5ms and 30ms. After 30ms, the curve rises to 75kN and quickly enters the descending section. This change in the curve is that the FH5 member was subjected to an axial force of 200kN. The member suffered severe local concrete damage and spalling at the beginning of the impact process.

**Fig 5:**
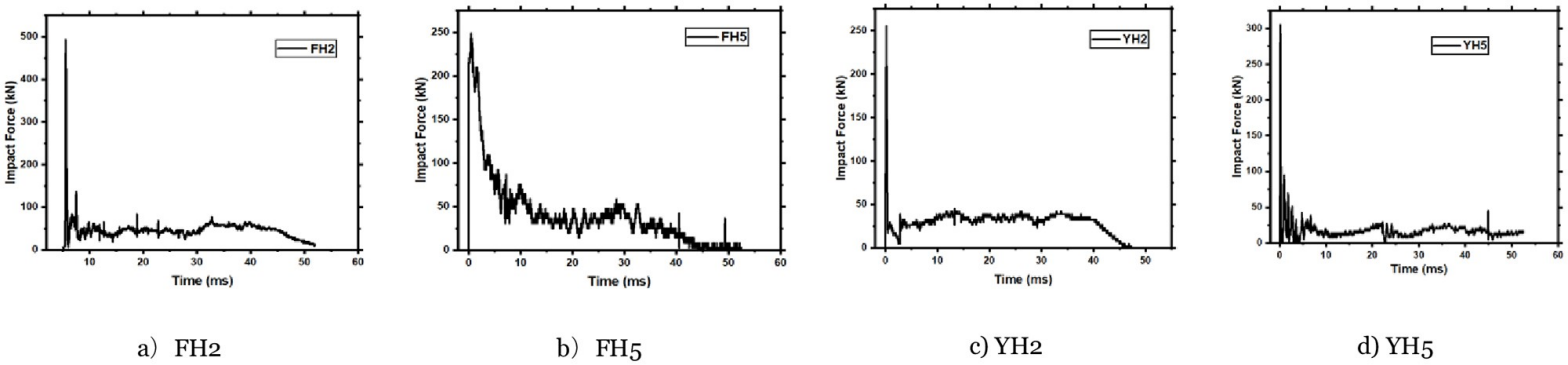
The time-history curve of the impact force of test member. a) FH2, b) FH5, c) YH2, d) YH5.

The overall bearing capacity of the member decreased significantly. After the longitudinal steel bar and stirrup bear the external load, the member’s bearing capacity increases quickly, so the curve appears to rise. To sum up, the impact force curve of an FH2 member can be divided into three stages, and the duration of the impact force curve of an FH2 member is shorter than that of an FH5 member. After the axial force is loaded, the impact force curve of the FH5 member only appears in two stages the peak section and the drop section. As presented for circular members, the peak impact force for all specimens is concentrated between 249kN to 486kN during (<4ms); the impact time history curves are shown in Fig 5. According to the impact force trend, the impact force curve can be divided into the peak, plateau, and unloading stages. Among them, the peak point occurs when the drop hammer is in contact with the member due to the massive difference in speed between the hammer and the concrete members, as shown in Fig 4. At this stage, the member’s deformation is minimal, and support is not entirely provided. Fig 5 shows that the peak impact force in member YH2 (258kN) is lower than that of specimen YH5 (304kN). Soon after, the peak impact stage ends when the impactor (hammer) and the RC members in the contact point have the same impact velocity. Then, the impact force trend entered the plateau stage. In this stage, the impactor fluctuated at the same speed as the RC member. In other words, the ability to stop the impactor is primarily attributed to the member’s lateral stiffness (or resistance) during this stage. After the plateau stage, the impactor and specimen have a different manner of motion [24]. This stage finishes after releasing all the energy stored in the hammer-member system.

On the other hand, it can be observed from Fig 5 that the peak impact force for specimen YH2 is 258kN, which is less than the peak impact force of member YH5. That is attributed to the existing axial load that causes an increase in concrete stiffness. Eventually, members’ resistance with an axial load for circular cross-section members is more than those without an axial load in the early stage.

### 3.3 Effect of the axial force on the deflection time-history curve

**Fig 6:**
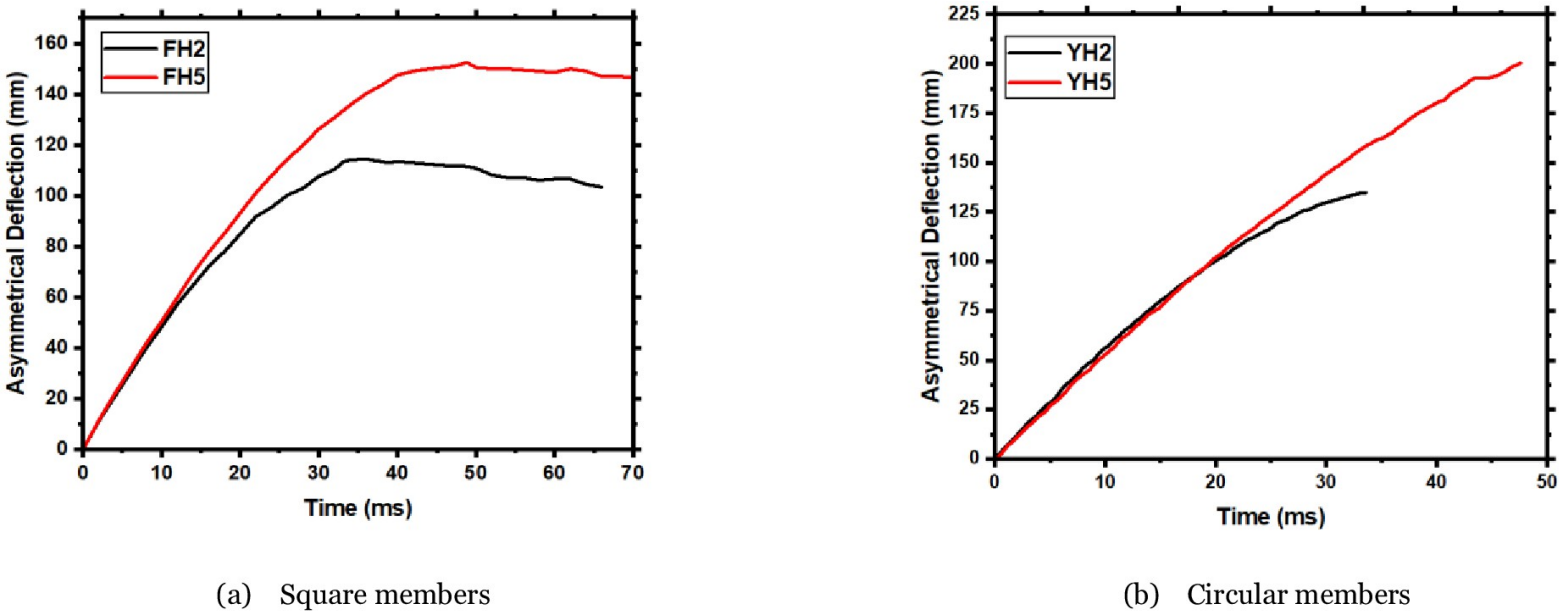
Deflection time history curve. a) Square members, b) Circular members.

Fig 6a is the time-history curve of the deflection at the impact point of the FH2 and FH5 members, and Table 2 gives the maximum deflection values of the members. The maximum deflection values of FH2 and FH5 are 114mm and 152mm, respectively, indicating that the maximum deflection value of members increases with adding the axial force. An increase of 31% indicates that axial force aggravates the damage degree of FH5 members, so the deflection value of FH5 members increases linearly. The curves of the two members, FH2 and FH5, have different degrees of rebound, indicating that the two members still have a small residual bearing capacity [25]. The deflection shows a slight rebound after reaching the maximum value, indicating that although all specimens have undergone different severe damage types, they still have a residual lateral Carrying capacity.

**Table 2:** Test members’ deflection curve.

Specimens Number	FH2	FH5	YH2	YH5
**Maximum deflection value/mm**	114	152	135	200
**Curved rebound segment**	exist	does not exist	exist	does not exist

Note: The YH5 member is severely damaged; use the latest deflection value as YH5’s maximum.

Furthermore, the maximum deflection of member FH2 occurred at 33ms, compared to 48ms in member FH5. Under different impact scenarios, the duration of the maximum deflection is greater than that of a member without axial load. That indicates the member was severely damaged due to the combined lateral impact and axial load. On the other hand, the axial load’s presence has greatly influenced the RC member’s performance. Fig 6b shows that member YH5 experienced a catastrophic failure in which the maximum deflection was around (200mm). Therefore, it can be concluded that the members subjected to a combined effect of asymmetrical transverse impact and axial load increases the brittle shear failure and thereby increases the member’s deformation.

## 4 Finite element analysis

Due to the small number of test members, it is difficult to effectively and completely analyze the axial force’s effect on RC members’ dynamic response. This section uses the ABAQUS 2020 software to establish RC members’ impact test simulation model. Choose appropriate material models, boundary conditions, and axial force application methods to lay the basics for subsequent analysis studies. ABAQUS software is widely used in research fields such as shock and explosion. It is easy to operate and has high simulation accuracy. It has great advantages in solving high-speed and temporary problems, mainly the Lagrangian algorithm, Euler algorithm, meshless algorithm, etc. The software has built-in hundreds of material libraries, which can simulate different materials freely and set parameters such as mutual contact properties, impact velocity, boundary conditions, and axial force of the model. The finite element model adopts a separate common node model. The author’s previous work adopts the definitions of impact body, material properties, and supports [26, 27]. When establishing a finite element model of an RC column, the size of the mesh density has a significant impact on the calculation accuracy of the model. Generally, the greater the mesh density, the higher the calculation accuracy [28]. Based on the above reasons, in this paper, the finer mesh is adopted for the unit of the impact point position, and more accurate data can be obtained. In ABAQUS, boundary conditions define how a structure interacts with its surroundings and play a critical role in determining the behavior of the structure under load. Various types of boundary conditions can be used in ABAQUS. This study used two types of boundary conditions for the members fixed and roller boundary conditions. Fixed boundary conditions prevent a structure from moving in a particular direction or around a certain axis. In ABAQUS, this is done by constraining the degree of freedom (DOF) in the relevant direction. In this study, members without axial force constrain all their DOFs (i.e., translational and rotational) in all three directions.

**Fig 7:**
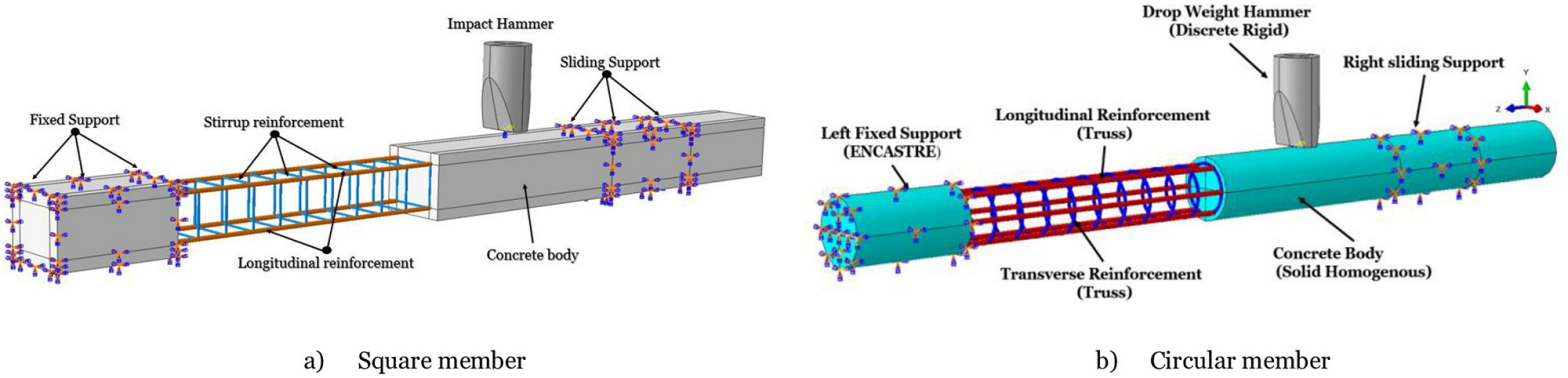
Modeling assembly. a) Square member, b) Circular member.

On the other hand, roller boundary conditions allow a structure to move freely in one direction but prevent it from moving in any other direction or rotating around any axis. This is accomplished by constraining all DOFs except one in the desired direction. For members with axial force, one side has fixed and others with roller boundary condition, to model the members on a roller support that can only move horizontally, constrain its vertical and rotational DOFs but leave its horizontal DOF unconstrained. Boundary conditions, loads, contact definition, strain-rate effects, mesh density, and convergence analysis calculations are mentioned in [19, 27]. Fig 7 shows the modeling assembly for a) square and b) circular members.

### 4.1 Application of axial force

**Fig 8:**
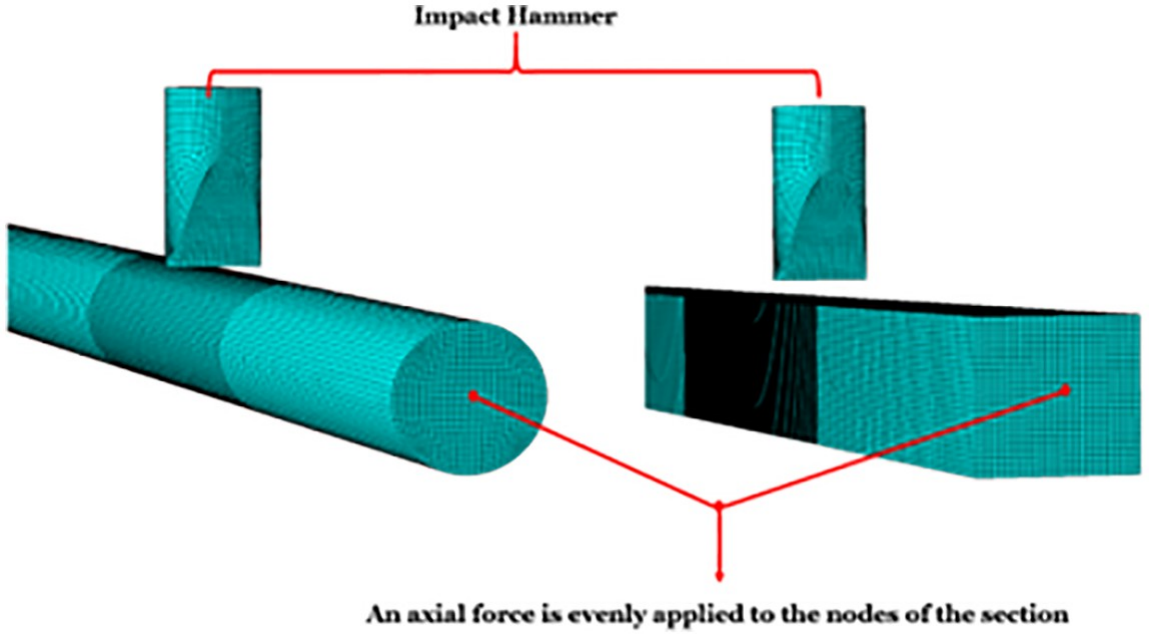
Axial force loading surface.

Thilakarathna et al. [29] proposed a feasible method of applying axial force when simulating the impact process of RC members. As shown in Fig 8, the axial force is evenly applied to the nodes of the section, and the calculation formula of the axial force at the nodes of the loaded section is shown in Eq (1).

f=Fn (1)

Where: *f* is the nodal axial force of the loaded section; *F* is the axial force; *n* is the total number of nodes of the loaded section. The axial force can be set in ABAQUS software using Module, Load, Create load, and concentrated force. The load keyword can define the applied direction of the axial force, and the defined keyword can define the magnitude of the axial force.

### 4.2 Verification of the finite element model

To verify the applicability and correctness of the selection of parameters in the model, a square and circular sections reinforced concrete member’s model was established and simulated using the test data of members.

### 4.3 Failure modes of members

**Fig 9:**
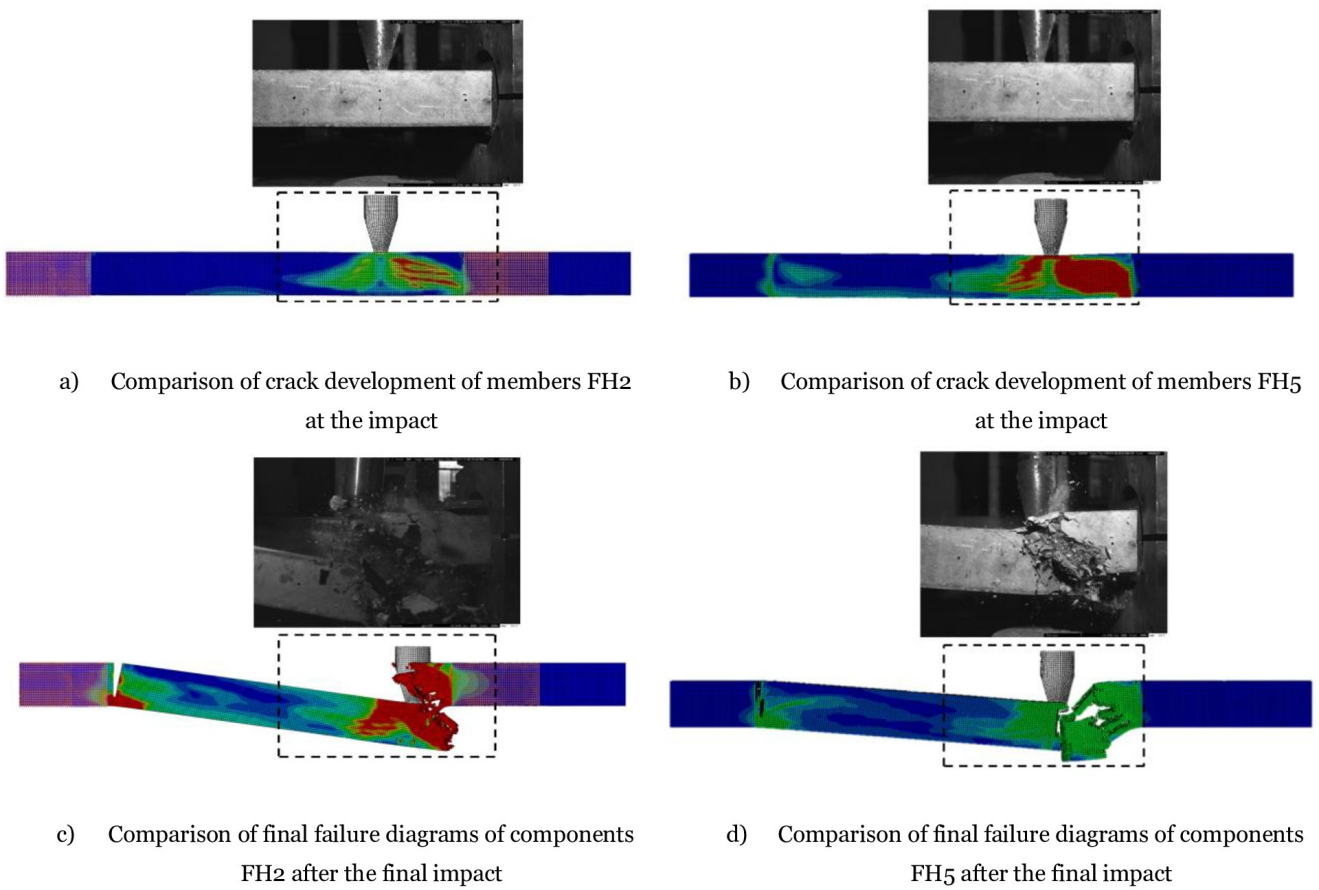
Failure modes of square member. a) Comparison of crack development of members FH2 at the impact, b) Comparison of crack development of members FH5 at the impact, c) Comparison of final failure diagrams of components FH2 after the final impact, d) Comparison of final failure diagrams of components FH5 after the final impact.

The crack development diagram of the RC member can be obtained using the ABAQUS software. The crack development diagram of the member at the time of 1ms is selected for comparison with the test diagram, as shown in Fig 9a and 9b. It can be found that the finite element model can accurately simulate the actual crack development of the members, the local concrete damage is consistent with the test results, and the bending cracks at the lower edge of the member section and the shear cracks on the right side are consistent with the test results. The failure modes of both the test and simulation are shear failures. Accordingly, the test members’ failure process and the local concrete spalling were at the impact point, and the through-band formed by shear cracks. Observing the final failure comparison diagram in Fig 9c and 9d, the simulation results effectively simulate the spalling of concrete materials and the fracture of reinforcing steel materials. The above results show that the established finite element model can better simulate the failure state of RC members under asymmetrical lateral impact and axial force.

**Fig 10:**
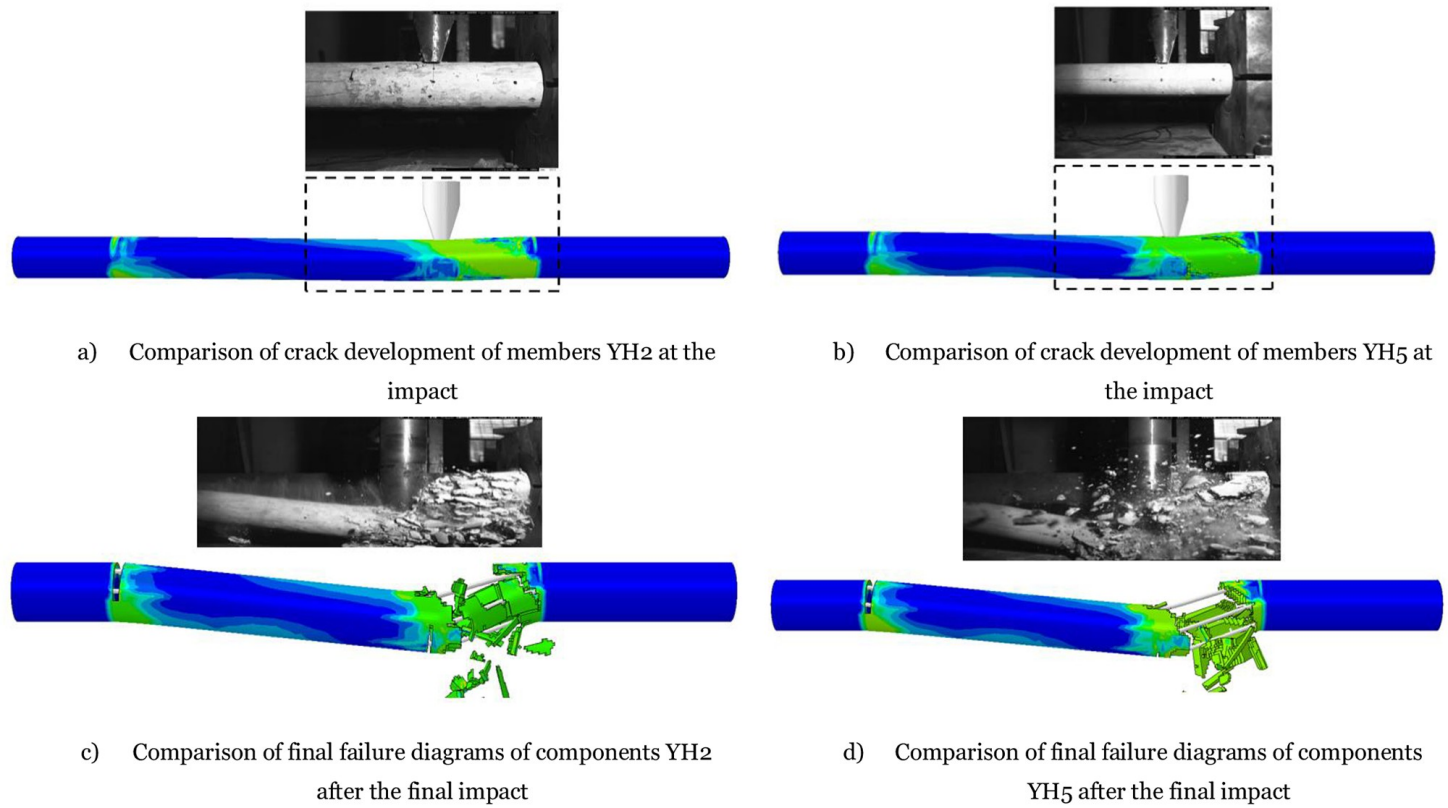
Failure modes of the circular member. a) Comparison of crack development of members YH2 at the impact, b) Comparison of crack development of members YH5 at the impact, c) Comparison of final failure diagrams of components YH2 after the final impact, d) Comparison of final failure diagrams of components YH5 after the final impact.

In summary, the comparison between the experimental results and the numerical analysis presented in Fig 10a and 10b indicates that the latter provides a reasonably accurate representation of the damage patterns observed in the circular members. The analysis predicts inclined damage to the concrete at 0.4ms, the initial stage of the damage process. This damage is followed by the appearance of main diagonal damage on both sides of the impact point, which develops gradually over time. The local failure observed at 1.6ms is caused by the crushing and spalling of concrete near the impact point, consistent with the experimental failure process. Furthermore, the analysis predicts that diagonal damage on the right side of the member extensively broadens at 4ms, a critical stage in the failure process. This is followed by catastrophic shear failure after 10ms, which causes the member to split into two pieces, as shown in the final failure mode presented in Fig 10c and 10d. Overall, the numerical analysis provides valuable insights into the damage process of the circular members, which can help improve the design and performance of such structures under impact loading.

### 4.4 Time-history curves of impact force and deflection

**Fig 11:**
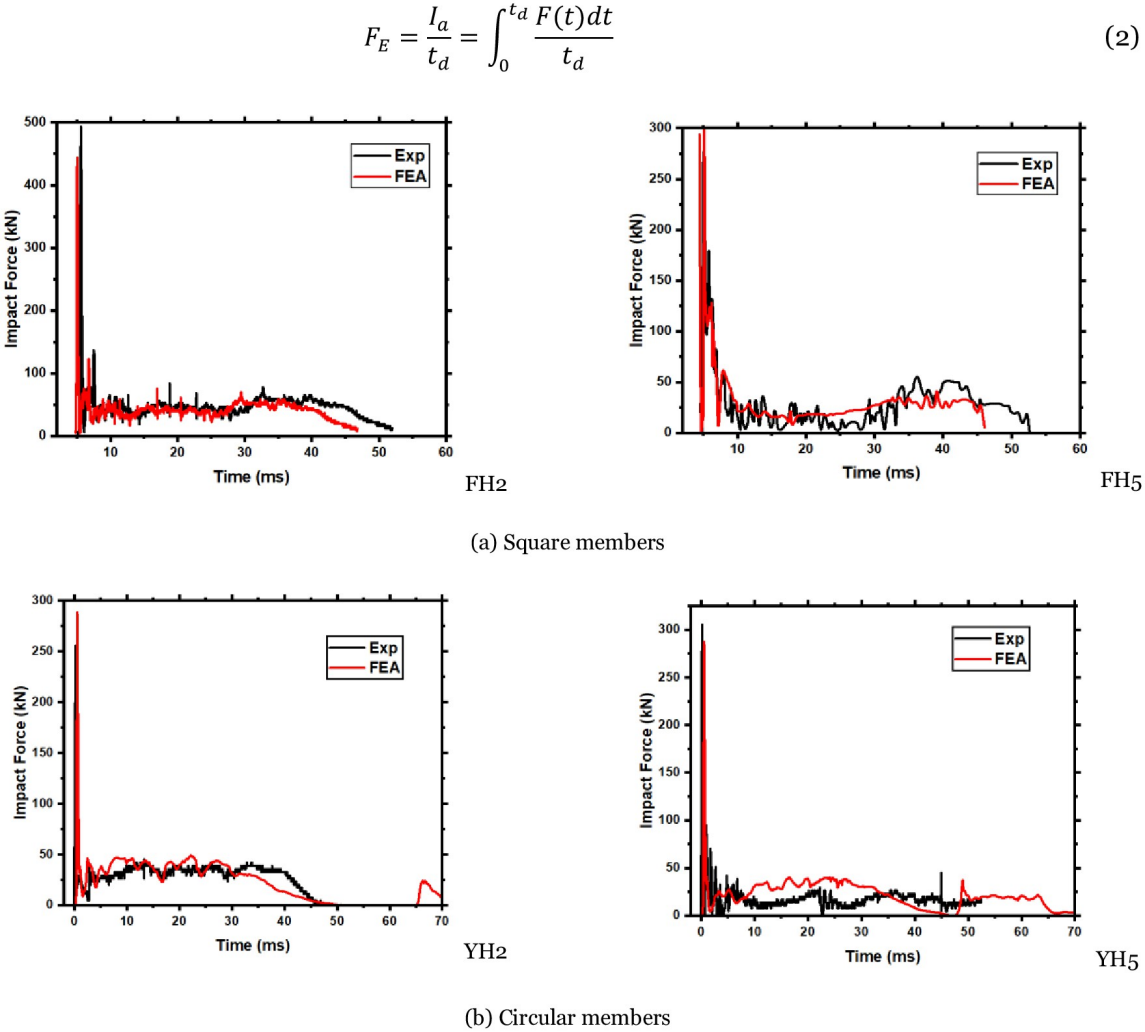
Comparison of simulated and experimental values of impact force time-history curve. (a) Square members, (b) Circular members.

The comparison between the simulated value and the experimental value of the impact force time-history curve for the square member is shown in Fig 11a. Overall, the simulated and experimental values of the impact force time-history curve only appear in the peak and descending segments. The curve shows small fluctuations after the peak segment. This phenomenon may be because the stirrups and longitudinal bars in the test members are connected by wire binding. At the same time, the finite element model adopts the common node model. As a result, the strength of the connection point is lower than that of the test member, and some steel joint elements are deleted after failure, resulting in a brief drop in the bearing capacity of the member and fluctuations in the impact force curve [20]. The initial peak disparity of the circular component can be attributed to various factors, including the load cell’s positioning and extensive concrete crushing at the impact point. However, a satisfactory correlation between the experimental outcomes and the finite element (FE) simulations was observed for the first impact peak and overall impact shape of the circular members, as illustrated in (Fig 11b).

Additionally, the models generated by the ABAQUS software exhibited accurate predictions for the essential features of the impact force curve, namely the mean impact force (Pm) and impact duration (Td). The mean impact force, regarded as a pure pulse discussed in prior studies [30], is expressed by Eq (2). The total contact duration between the hammer and the reinforced concrete (RC) members has been defined in earlier research [31].


$$F_{E}=\frac{I_{a}}{t_{d}}=\int_{0}^{t_{d}}\frac{F\left(t\right)dt}{t_{d}}$$ (2)

**Fig 12:**
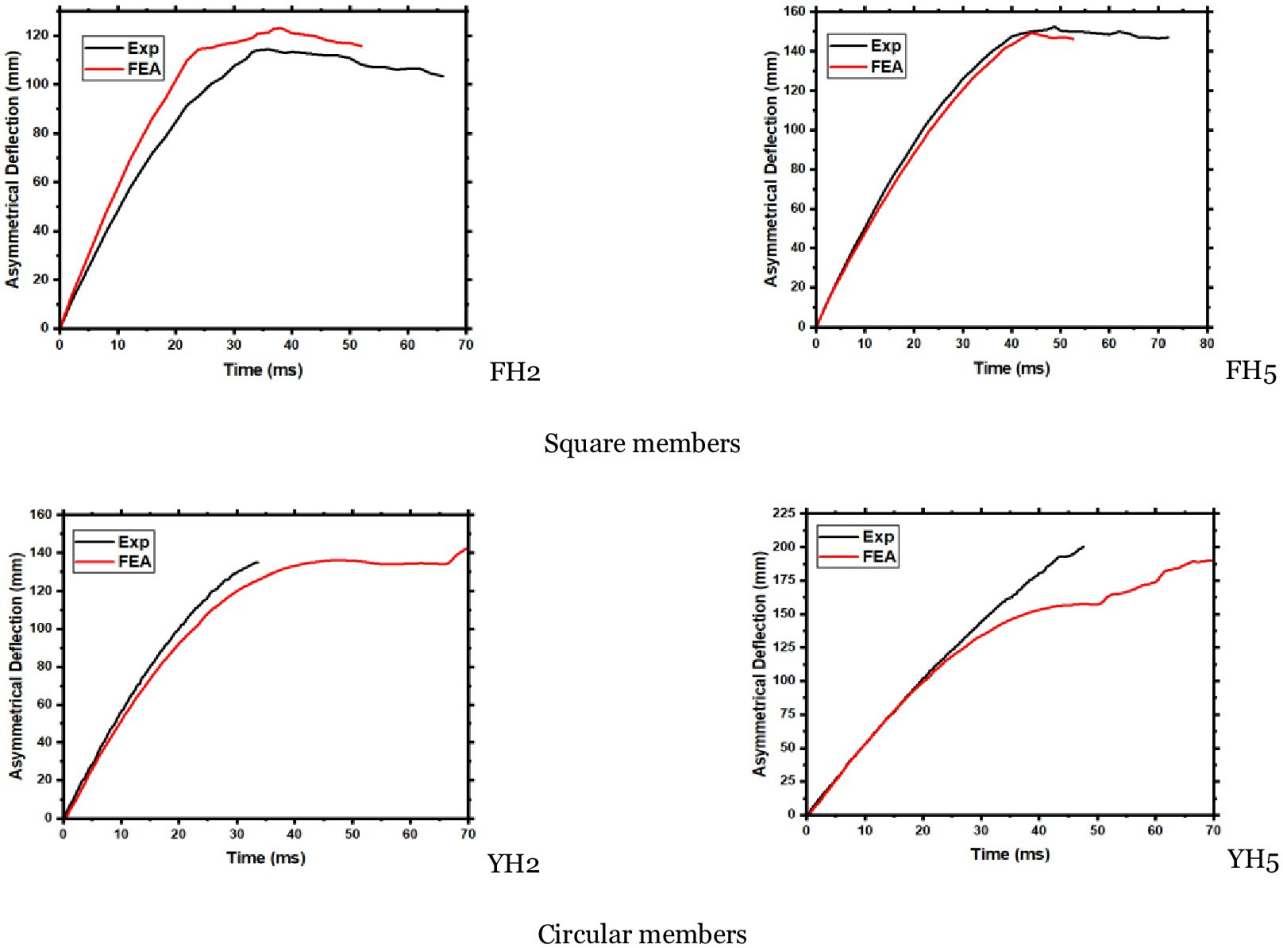
Comparison of simulated and experimental values of deflection time-history curve. Square members, Circular members.

The deflection-time history curves of the modeled members in Fig 12 at a distance of 200 mm from the right support demonstrate that the simulated values are consistent with the experimental values. The validation of the experimental tests is further elaborated in Table 3, which indicates that the maximum error for circular members whose impact duration exceeds 30% is within 15%. It should be noted that for a member that experienced complete failure during the experiment, no data was recorded beyond 60ms. In contrast, the simulation data recorded rebound values of the impactor until it stabilized after 70ms. The aforementioned errors include assumptions made during the model establishment, such as the rigidity of the impact body, zero heat dissipation of material deformation, and the presence of friction force [32].

**Table 3:** Compares the main results collected from FE models and experimental outcomes.

No.	Peak asymmetrical impact force *P*_max._ (kN)		Diff. %	Duration *T*_d_ (ms)		Diff. %	Mean asymmetrical impact force *P*_m_ (kN)		Diff. %	Maximum asymmetrical deflection *δ*_*max*._ (mm)		Diff. %
	Exp.	FEA		Exp.	FEA		Exp.	FEA		Exp.	FEA	
FH2	493	442	10.3	49.3	47.1	4.4	52.4	48.4	7.6	114	125	8.8
FH5	286	305	6.2	52.5	46.6	11.2	37.5	32.1	14.4	152	150	1.3
YH2	258	287	10.1	46.5	72	35.4	43.5	38.4	11.7	135	141	4.2
YH5	304	285	6.2	54	72	25	26.6	32.2	17.4	200	188	6

To sum up, by comparing the members’ failure form, impact force, and deflection time-history curve, the finite element model can more accurately simulate the impact process of the RC members under the action of axial force, and the calculated data can meet the requirements of subsequent analysis.

## 5. Parametric study

**Fig 13:**
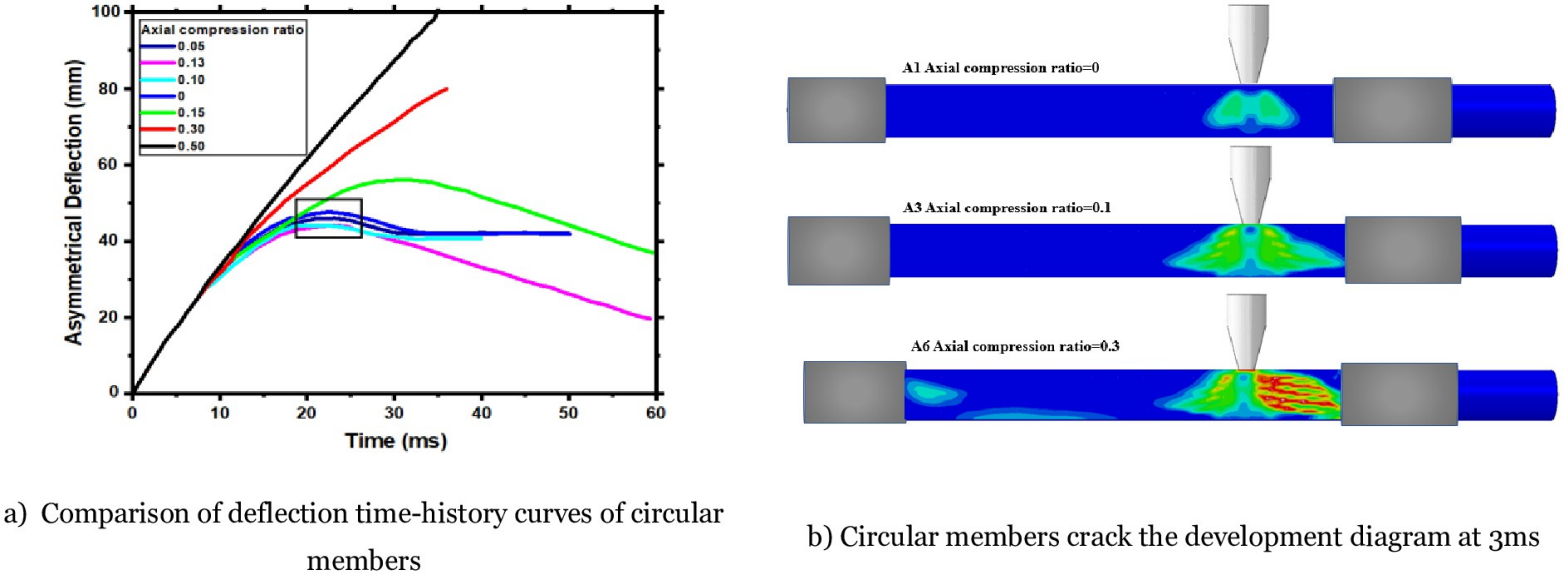
Comparison of deflection time-history curves crack and development diagram of circular members. a) Comparison of deflection time-history curves of circular members, b) Circular members crack the development diagram at 3ms.

The finite element simulation model of the impact process of the RC members is established, and the test data verify the applicability and correctness of the ABAQUS model. In this part, finite element software is used to simulate the impact process of RC members under different axial compression ratios, and the influence of axial force on the dynamic response of RC members is further studied. The main factors, such as preloaded axial force, maximum principal stress of concrete, shear crack penetration time, CFRP layers strengthening, and impact position, are analyzed to understand the impact of these parameters on the mechanical properties of RC members under asymmetrical impact loads. The impact test scenario of the RC members is analyzed in detail, and the axial force significantly affects the RC member’s dynamic response. However, during the test, the impact energy of the FH5 and YH5 members was large, and the two members were severely damaged. The concrete at the impact point is damaged and peeled off in a large area. The longitudinal steel bars and stirrups are broken, resulting in the rapid destruction of the two members, and it is challenging to collect test data accurately and comprehensively. The test found that the impact energy of the FH2 and YH2 members is small, the damage degree of the section is relatively light, and there is no large-area spalling of the concrete and fracture of the steel bar. The YH2 and FH2 can be used as a basic members to study the axial force’s effect on the RC member’s dynamic response. The ABAQUS software used the parameter to simulate the impact process of the selected member under the conditions of different axial compression ratios. More data were added to study the axial force’s specific effects on the RC member’s dynamic response. The RC member’s axial bearing capacity calculation methods are given in "Code for Design of Concrete Structures" [33], and the axial bearing capacity of the FH2 and YH2 members are 586.4kN and 543kN. The simulation and test calculation of the members found that when the axial compression ratio is greater than 0.3, the greater the axial force, the more serious the damage to the members. Fig 13 shows the deflection curve of the circular members when the axial compression ratio is 0.5, and the deflection develops linearly. The trend is consistent with the development trend when the axial compression ratio is 0.3. The axial compression ratio greater than 0.3 is less helpful for studying the favorable range of the axial force, so the maximum axial compression ratio of the parameter’s member is set to 0.3. The axial compression ratios of parameter members are shown in Table 4.

**Table 4:** Axial compression ratio of members.

Numbering	A1	A2	A3	A4	A5	A6
**Axial compression ratio**	0	0.050	0.10	0.13	0.15	0.30

The finite element simulation results of A1 to A6 members are as follows, and the results show that different magnitudes of axial force will affect the dynamic response of RC members.

### 5.1 Effect of axial force on members’ asymmetrical deflection

The asymmetrical deflection time-history curves of circular members A1-A6 are shown in Fig 13a. The deflection of the RC members under different axial compression ratios does not change significantly before 0.08s, and the shape and value of the curve are the same. After 8ms, the shape and value of the deflection time-history curves of different axial forces began to appear different. The axial force has no significant effect on the deflection development of the members at the beginning of the impact process (t < 0.008s). The crack development diagram is shown in Fig 13b. Combined with the Figure, it can be found that there is no significant difference in the development of cracks among the A1, A3, and A6 circular members at this moment. Bending and shear cracks appear in all the members, and the length and width of the cracks are the same. It can be inferred that there is no overall failure of the circular RC member at this stage. The axial and lateral bearing capacity of the members does not decrease significantly. The deflection value is mainly the elastic deformation of the members; when the axial compression ratio>0.05, the maximum deflection value of the member begins to decrease. The axial compression ratio = 0.10, and the maximum deflection value of the A3 is reduced to (43mm), which is about 8% lower than that of the A1 (47mm). The axial compression ratio is 0.13, and the maximum asymmetrical deflection value of the A4 does not continue to decrease, indicating that the axial compression ratio is a limit value that affects the member’s dynamic response. Then, the axial compression ratio is in the range of 0.05~0.13; the greater the axial force, the smaller the maximum deflection value of the component. The decrease in the maximum deflection value indicates that the impact resistance of the circular member becomes better. When the axial compression ratio = 0.30, the member deflection increases linearly with the impact process. The curve has no rebound section, indicating that the member is severely damaged and the residual bearing capacity is extremely small.

Observing (Fig 13a), there is a good range for the axial compression ratio to keep the maximum deflection value of the member smaller (marked by the black box): 0.050~0.13. When the axial compression ratio is within this range, the greater the applied axial force, the smaller the maximum deflection of the member. This indicates that the axial force has both adverse and beneficial effects on the dynamic response of the RC member, and there is a good range that can improve the impact resistance of the member.

### 5.2 Effect of axial force on members’ asymmetrical lateral impact

**Fig 14:**
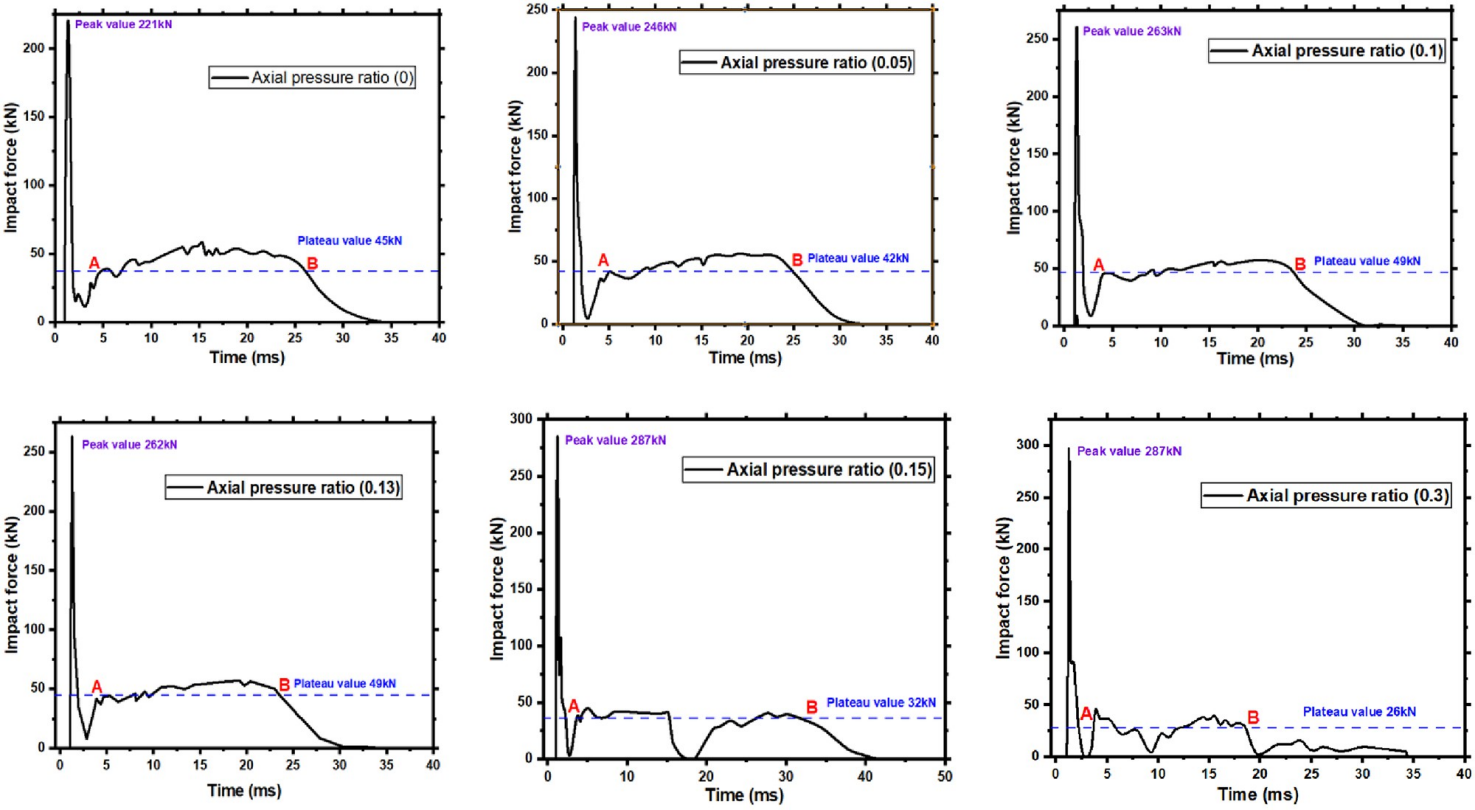
The time-history curve of the impact force of members.

The impact force time-history curves of A1-A6 members are shown in Fig 14, according to the definition of the plateau stage of the impact force curve in [19, 34]. Table 5 gives the peak and plateau values of the impact force of the six members. The impact force curve of A1-A4 members can be divided into three stages. Peak, plateau, and descent stage. It can be found that the impact force curves of members A5 and A6 fall and then rise after the first plateau and the second plateau stage appears. Therefore, the impact force curves of the two members are defined as four stages: peak, major plateau, minor plateau, and descent stage. The greater the axial force, the greater the peak value of the impact force of the member. The peak value of the impact force of the A6 member is 43% higher than that of the A1 member. Since the peak impact force is directly related to the contact stiffness, other things being equal, it can be inferred that the axial force changes the contact stiffness at the impact point of the member. Different magnitudes of axial force have different influences on the plateau value of the impact force curve of the member and also show a good range of the influence of the axial force.

**Table 5:** Peak impact force and plateau value of B1-B6 members.

No.	Axial compression ratio	Peak value/kN	Plateau value/kN	No.	Axial compression ratio	Peak value/kN	Plateau value/kN
**A1**	0	209	47	A4	0.13	265	50
**A2**	0.05	249	49	A5	0.15	275	35
**A3**	0.1	259	51	A6	0.3	297	27

A good range for the axial force’s influence on the impact force’s plateau value exists, consistent with the good range found in the deflection time-history curve. Eventually, it can be seen that the magnitude of the axial force is positively correlated with the peak value of the impact force. The ideal range is 0.13 to 0.50. The impact of the axial force on the member’s dynamic response is undesirable when the axial compression ratio exceeds the desirable range.

### 5.3 Maximum principal stress of concrete at the impact point

**Fig 15:**
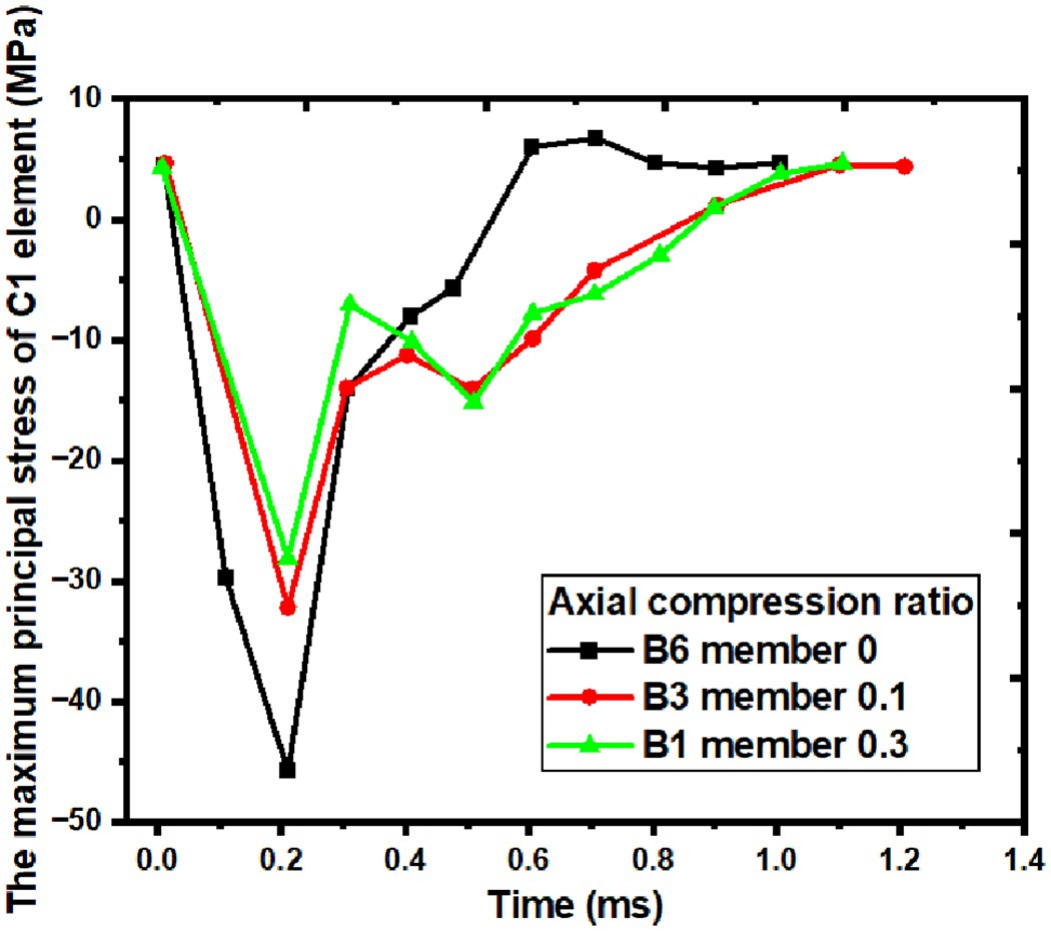
Maximum principal stress diagram for concrete element C1.

The large-area failure and peeling of local concrete occur at the impact point, and the stress of concrete directly affects the degree of damage. To study the stress of local concrete, the characteristic unit of concrete C1 is selected as the research object. In the test and finite element simulation, it is found that the earlier the shear crack penetrates, the earlier the member will fail, so the characteristic elements of concrete C2 and C3 are selected to study the shear crack penetration. The maximum principal stress curve of the concrete C1 element is extracted using the built-in History-Element function of the software, as shown in Fig 15. The numerical value of the curve in the Figure is negative, indicating compression. Observing Fig 15, the maximum principal stress curve of element C1 can be divided into two stages: the peak stage and the drop stage [35]. The peak stage is when the impact body contacts the member to the curve’s maximum value, representing the concrete member’s maximum stress value. The descending stage is the range of the curve from the maximum value to zero, which represents the change of stress after the concrete element begins to fail. In the curves of members A1, A3, and A6, the moment when element C1 reaches the maximum stress is about 0.2ms, which means that as soon as the impact body contacts the member, the concrete at the impact point reaches the maximum stress instantaneously.

As shown in Table 6, the peak value of the A1 and A3 members is 30MPa and 32MPa, and the change is small compared with the peak value of the A1 member. The peak value of the A6 is 46MPa, which is more than 50% more than A1’s peak compression. The stress quickly reduces after 0.2ms when the concrete part is damaged and the ascending stage of the curve begins. In the descending portion of the three curves, there are variations. Stress is distributed differently during the impact process due to the concrete collapse. As shown in Table 6, the maximum principal stress of the C1 member in the A6 member with the axial compression ratio of 0.3 reaches the maximum value. It then drops to zero in the shortest time, only 0.4ms. This phenomenon is because the maximum principal stress of the C1 member in the A6 member is the largest, leading to the concrete’s faster collapse. The curve zeroing time of the A3 member is less different from that of the A1 member.

**Table 6:** Comparison of peak values of maximum principal stress curves.

Component number	Axial compression ratio	Peak/MPa	Time peak-zero (ms)
**A1 member**	0	30	0.8
**A3 member**	0.1	32	0.9
**A6 member**	0.3	46	0.4

To sum up, when the axial compression ratio is 0.3, the axial force increases the maximum principal stress of the local concrete, which leads to the faster collapse of the concrete at the impact point. The concrete and reinforcement then fail to work together, reducing the member’s axial and lateral bearing capacity at the beginning of the impact process.

### 5.6 Shear crack penetration time

**Fig 16:**
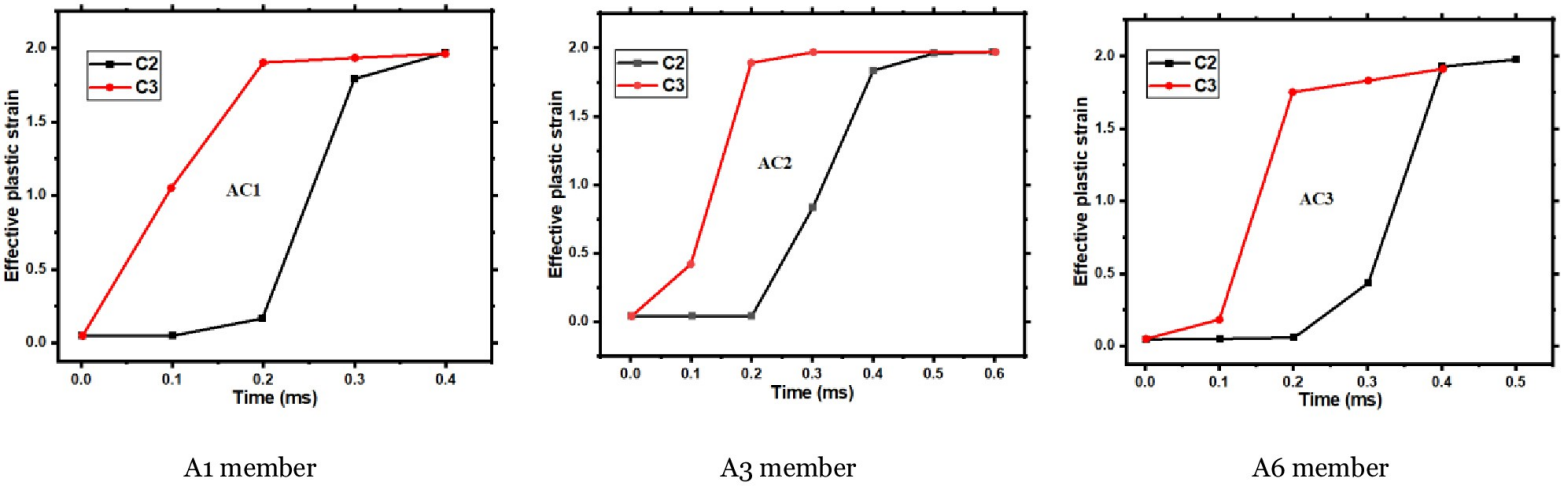
The effective plastic strain development diagram of elements A2 and A3. A1 member, A3 member, A6 member.

Generally speaking, the faster the penetration speed of shear cracks, the earlier the shear cracks penetrate, and the easier it is for members to fail catastrophically under impact. Use the ABAQUS software’s effective plastic strain (EPS) function to obtain the effective plastic strain curves of the C2 and C3 elements Fig 16. The effective plastic strain of the element reaches 2, and the concrete cracks at this position. The direction of the shear cracks in the member in the finite element simulation is developed from the C2 element to the C3. The enclosed area by the C2 and C3 element strain curves (*AC*_1_, *AC*_2_, *and AC*_3_) represents the time difference when the two curves reach the same strain. Then, the smaller the enclosed area, the faster crack development.

As shown in Table 7, the areas of (*AC*_1_, *AC*_2_, *and AC*_3_) regions calculated by Drawing software are 20, 23, and 18, respectively. The failure time difference between C2 and C3 elements is the longest for the A3 member and the shortest for the A6 member. The smaller the time difference, the faster the crack penetration. The results show that the penetration speed of the shear crack in the A3 member is the slowest, the penetration time is the latest, and the member does not fail prematurely, ensuring the member’s overall stress. The shear crack in the A6 member has the fastest penetration speed and the earliest penetration time, resulting in serious damage to the member and linearly increasing the deflection of the A6 member.

**Table 7:** Shows the *AC* area and the time difference between elements C2 and C3.

Component number	Axial compression ratio	*AC* area	C2, C3 element failure time difference (ms)
**A1 member**	0	20	0.2
**A3 member**	0.1	23	0.3
**A6 member**	0.3	18	0.1

To summarize, the axial force determines the penetration speed of shear fractures. When the axial compression ratio is 0.05~0.13, shear crack growth is sluggish, and the creation of the penetration failure zone is delayed. When the axial compression ratio exceeds 0.13, the shear fracture develops more quickly, and the penetration failure zone develops earlier.

## 6. Adding CFRP layers to RC members with axial force

### 6.1 Effect of CFRP layers thickness strengthened components

**Fig 17:**
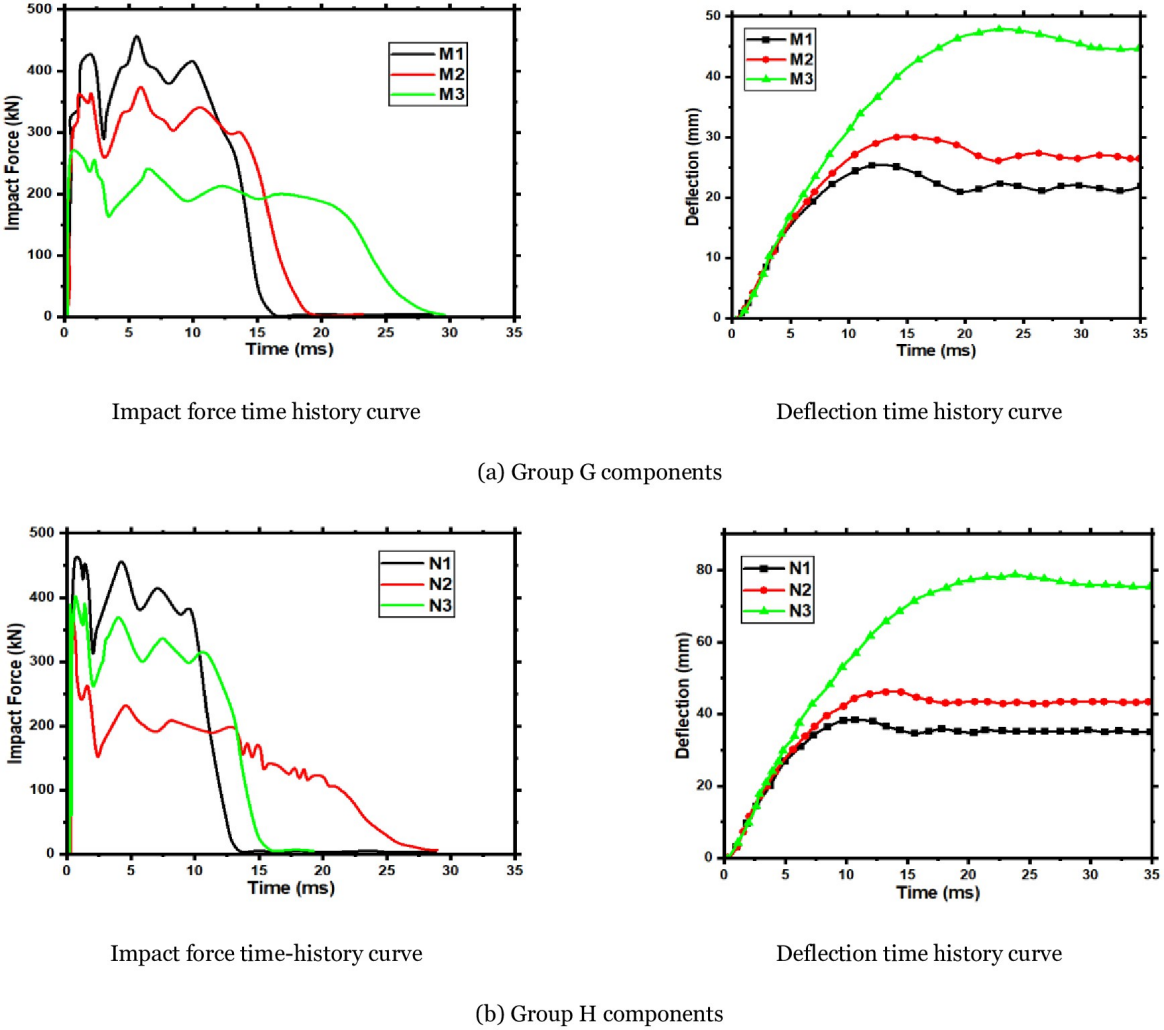
Simulation results of group H and G components. (a) Group G components, (b) Group H components.

In this section, the main factors, such as impact velocity, CFRP layers thickness, and preloaded axial force, are analyzed to understand the impact of these parameters on the mechanical properties of RC members with CFRP layers under asymmetrical impact loads. This section divides the components into two groups, M and N. The impact height of the components in group M is 3.0m, and in group N is 5.0m. Three different CFRP Layers thicknesses of one, four, and six layers are set for each group of components, and an axial preloading force of 200kN is applied to each component. The mass of the impact body is 270kg. Table 8 shows the simulation conditions and results of Groups M and N. The dynamic response results of the two groups of components are shown in Fig 17.

**Table 8:** Group M and N components simulation conditions and calculation results.

Specimen number	CFRP layers thickness/mm	Impact height/m	Axial compression/kN	Impact body mass/kg	Impact duration/s	Peak impact force/kN	Impact force plateau value/kN	Maximum deflection/mm	Damage condition
**M1**	One layer	3.0	200	270	0.0143	272	203	34	deflection
**M2**	Four layers	3.0	200	270	0.0097	385	324	22	deflection
**M3**	Six layers	3.0	200	270	0.0092	465	404	18	deflection
**N1**	One layer	5.0	200	270	0.0207	366	201	60	deflection
**N2**	Four layers	5.0	200	270	0.0118	435	320	35	deflection
**N3**	Six layers	5.0	200	270	0.0098	467	400	30	deflection

Combining the curves shows that when the impact height is the same, the impact force’s peak and plateau value greatly improve with increased CFRP layers’ thickness. Nevertheless, the duration of the peak stage is unchanged, while the duration of the plateau stage is significantly reduced. The components’ maximum and residual deflection decrease with the CFRP layers’ thickness increase. For the members of group M, the deformation degree of the components becomes smaller with the increase of the thickness of the CFRP layers. In contrast, the impact height of the components of group N is larger when the CFRP layer thickness is four layers. The components were cracked at the impact position and the right support near the impact point. Increasing the CFRP layers thickness to four and six layers deformed the components, but the degree of deformation became smaller. Consequently, the CFRP layers’ thickness significantly affects the impact resistance of RC members. Increasing the CFRP layer thickness increases the impact resistance of the components.

### 6.2 Effect of impact position on components

**Fig 18:**
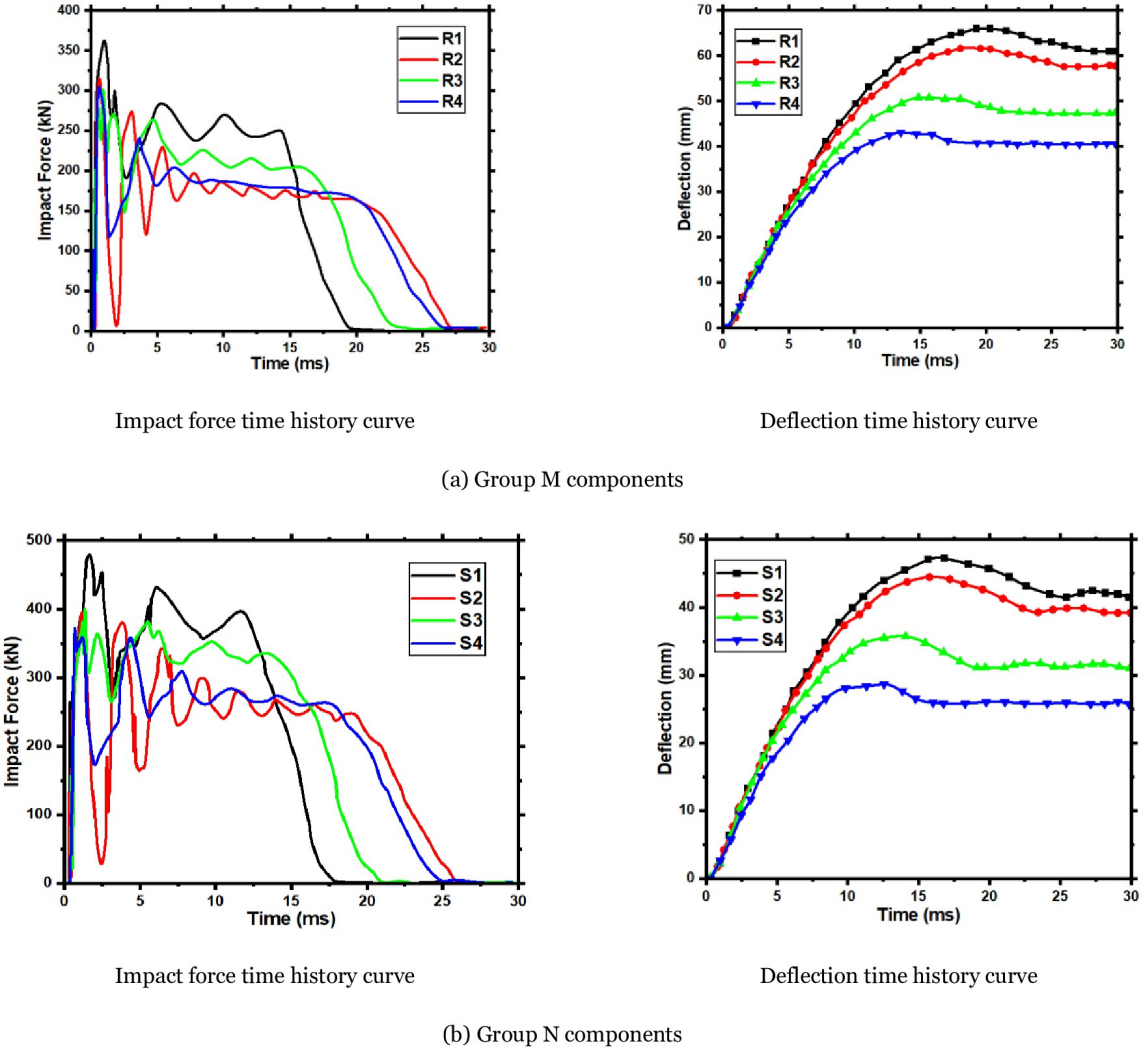
Simulation results of group M and N components. (a) Group M components, (b) Group N components.

In this part, two groups of members, R and S, are set up in which the CFRP layers thickness of the R group component is one layer, and the S group component CFRP layers thickness is four layers. The impact position of each group is 150mm, 250mm, 350mm, and 450mm from the support, respectively. Meanwhile, 450mm is the mid-span position of the member’s effective length. The impact height is 3m, the mass of the impact body is 270kg, and an axial preloading force of 200kN is applied to each component. The simulation conditions and results of Group R and S components are shown in Table 9, and the dynamic response results of the two groups of components are shown in Fig 18.

**Table 9:** Group M and N component simulation conditions and calculation results.

Specimen No.	CFRP layers thickness/mm	Impact height/m	Axial load/kN	Impact position (mm)	Impact duration/s	Peak impact force/kN	Impact force plateau / kN	Maximum deflection/mm	Damage condition
**R1**	one layer	5.0	200	150	0.014	367.6	221	66.5	deflection
**R2**	one layer	5.0	200	250	0.016	314.7	190	62.6	deflection
**R3**	Two layers	5.0	200	350	0.018	303.4	162	51.8	deflection
**R4**	Two layers	5.0	200	450	0.019	305.4	157	43.8	deflection
**S1**	Four layers	5.0	200	150	0.010	475.6	344	47.5	deflection
**S2**	Four layers	5.0	200	250	0.011	402.4	308	44.7	deflection
**S3**	Four layers	5.0	200	350	0.013	408.5	248	36.7	deflection
**S4**	Four layers	5.0	200	450	0.014	385.2	239	28.8	deflection

Based on the Figure, combined with the curve, it can be concluded that as the impact position gets further away from the support, the peak impact force decreases and then increases. The plateau value of the impact force decreases gradually. The impact duration also increases. Simultaneously, the maximum and residual deflection of the impact position of the component also increases gradually. During the mid-span impact, the impact force plateau value reaches the minimum, and the component deflection reaches the maximum. Compared with the impact at 150mm distance from the support, the impact force plateau value of R group components during mid-span impact is reduced by 29.4%, and the maximum deflection of components increases by 53.3%. Compared with the impact at 150mm from the support, the impact force plateau value of the S group component is reduced by 30.6%, and its maximum deflection is increased by 65.0%. Strictly speaking, the mid-span is the most unfavorable position for RC components to resist lateral impact [18]. The farther from the mid-span, the stronger the impact resistance. It is also consistent with the conclusion put forward by Yousuf et al. [36–38] that "the deflection at the impact position at the 1/4 span length is smaller than that of the mid-span impact and can bear a larger load".

### 6.3 Effect of bending moment on components

**Fig 19:**
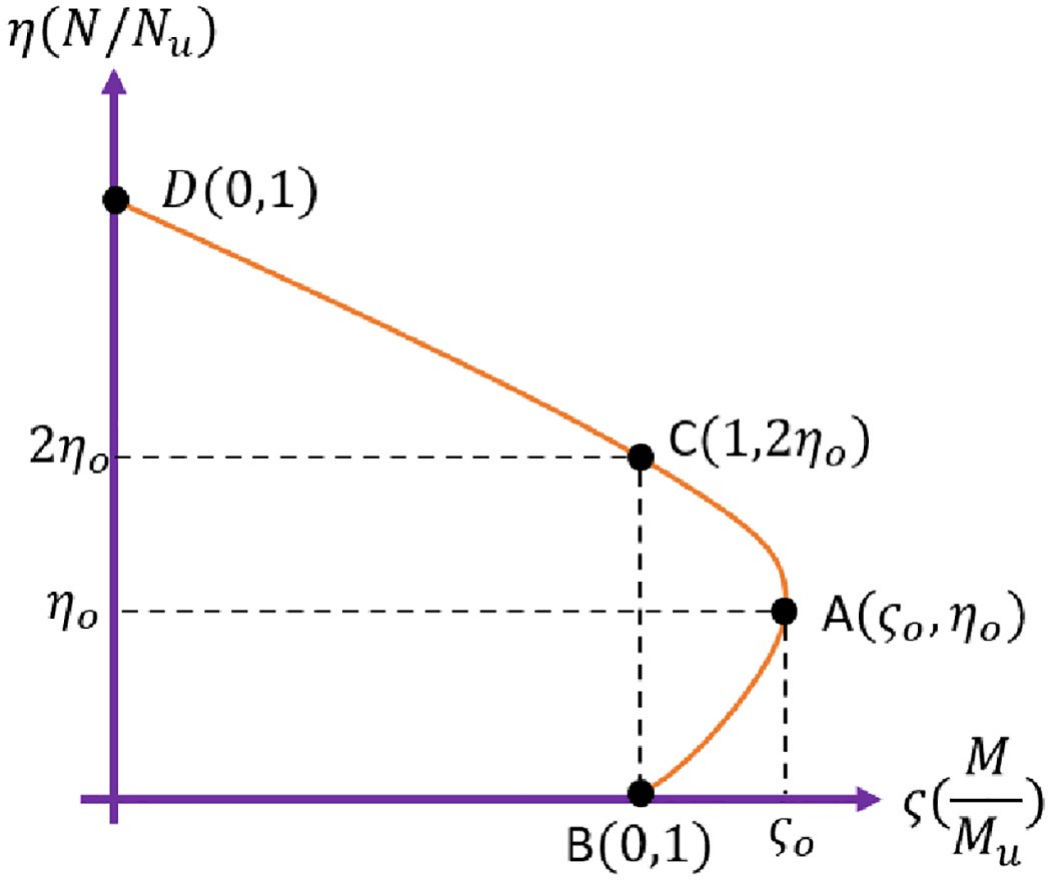
Typical axial force (N), bending moment (M) strength relationship curve.

The plastic limit bending moment decreases when the axial force increases to a certain value. However, it is still larger than that without the axial force. The variation trend of the plastic limit bending moments of each group of components can be explained by the bending moment (M) and axial force (N) strength relationship curve of CFRP-RC given in Fig 19 [39, 40]. When the ratio of the axial force N of the component to the axial bearing capacity *N*_*o*_ *is η* < *η*_*ο*_, with the axial compression ratio increase, the section’s bending moment can withstand increases. The plastic limit bending moment increases when *η*_*ο*_ < *η* < 2*η*_*ο*_, and the plastic limit bending moment begins to decrease with the increase of the axial compression ratio. At point A, the plastic limit bending moment will reach its maximum. The values of *ς*_*ο*_ *and η*_*ο*_ are affected by factors such as the strength of CFRP and concrete, the layer thickness, and the slenderness ratio of components. The greater the CFRP yield strength, the multi the CFRP layer thickness. The larger the slenderness ratio of the component, the smaller *ς*_*ο*_ *and η*_*ο*_. The stronger the concrete, the greater the *ς*_*ο*_ *and η*_*ο*_.

**Fig 20:**
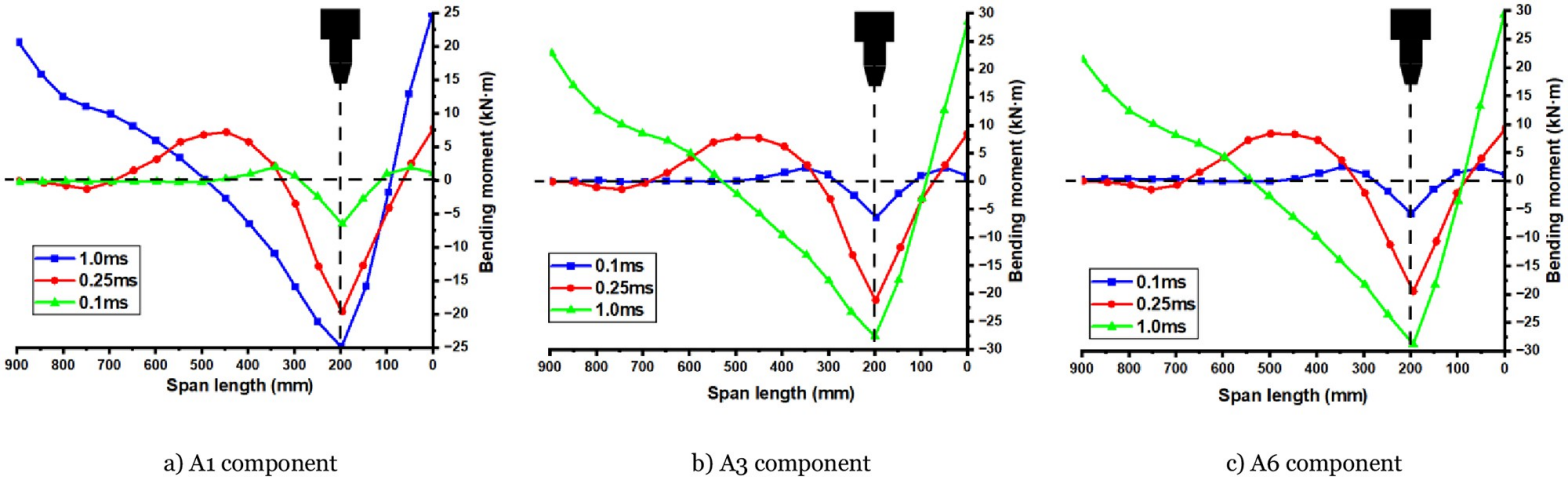
A1, A3, A6 bending moment variation. a) A1 component, b) A3 component, c) A6 component.

As depicted in Fig 20, the bending moment diagrams of three components (A1, A3, and A6) are presented under the influence of new factors (four layers of CFRP thickness, impact height of 5m, and axial compression ratios of 0, 0.1, and 0.3, respectively) at different times. The bending moment can be divided into two stages: the first stage corresponds to the peak impact force stage, and the second stage occurs after the peak. The first stage is discussed in Fig 20. During this stage, axial force does not affect the development of the bending moment distribution. At 0.1ms, the bending moment is symmetrically distributed on both sides of the section at the impact position because the stress wave has not yet been transmitted to the supports at the ends. As a result, the development of the bending moment on both sides is kept synchronous. At 0.25ms, the stress wave reaches the right support first due to its shorter distance, while the stress wave on the left continues to transmit to the left support. This leads to a positive bending moment on the right-side support and a lack of bending moment development in the range of 600mm to 900mm on the left span. At 1ms, the impact force reaches its peak value and begins to decrease, leading to peak values for the bending moment at the impact position and the left and right sides of the support. In the second stage, the bending moments of each component’s supports’ left and right sides remain unchanged.

In contrast, the bending moment of the component at the impact position remains unchanged for a while before decreasing. This suggests that the bending moment of each part remains constant during this period, largely due to the plastic yielding of the parts on both sides of the support and at the impact position. This asymmetrical lateral impact of CFRP-RC components under axial compression demonstrates bending deformation and failure characteristics. An appropriate amount of axial force can improve the plastic development ability of the entire component, increase each part’s dynamic plastic limit bending moment, and extend the duration of plastic development. However, if the axial force is too large, plastic development will be accelerated, and the damage will be more significant.

### 6.4 Effect on the energy absorbed by components

**Fig 21:**
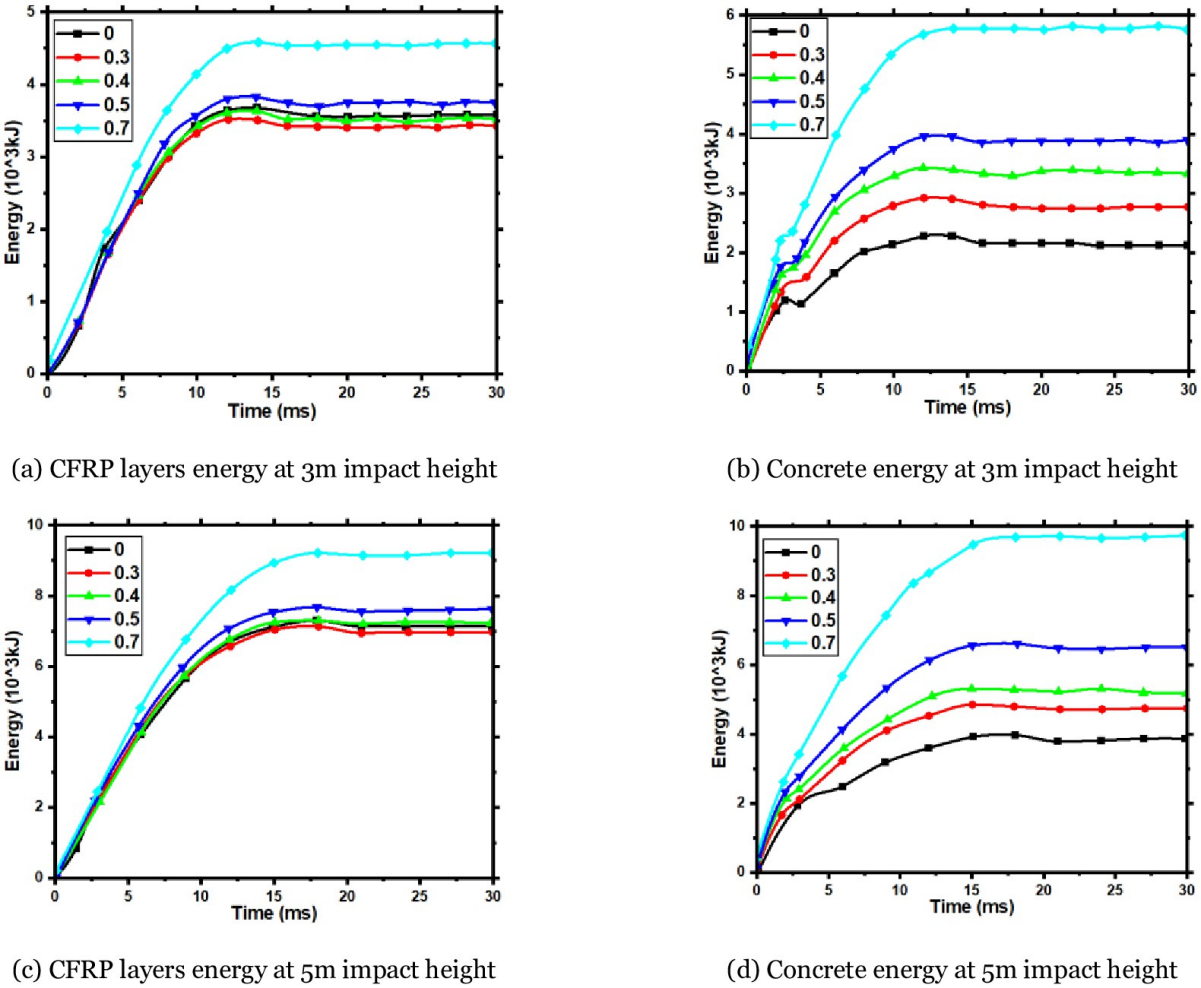
Energy variety of CFRP layers and concrete for each component. (a) CFRP layers energy at 3m impact height, (b) Concrete energy at 3m impact height, (c) CFRP layers energy at 5m impact height, (d) Concrete energy at 5m impact height.

The time history of the energy absorption of CFRP layers and concrete during the impact of each component of Group A at 3m and 5m impact heights is depicted in Fig 21. This Figure illustrates the sum of the kinetic and internal energy of the CFRP layers and concrete. It can be observed from Fig 21a and 21c that, regardless of the impact height of 3m or 5m, the axial compression ratio increases to 0.1. The energy of the CFRP layers is slightly lower than that in the absence of axial compression. However, when the axial compression ratio increases to 0.1, the energy is similar to that without axial force. As the axial compression ratio increases, the CFRP layers’ energy exceeds the state without axial force. Fig 21b and 21d) reveal that the concrete’s energy increases with the axial compression ratio.

### 6.5 Effect on the stress of components

**Fig 22:**
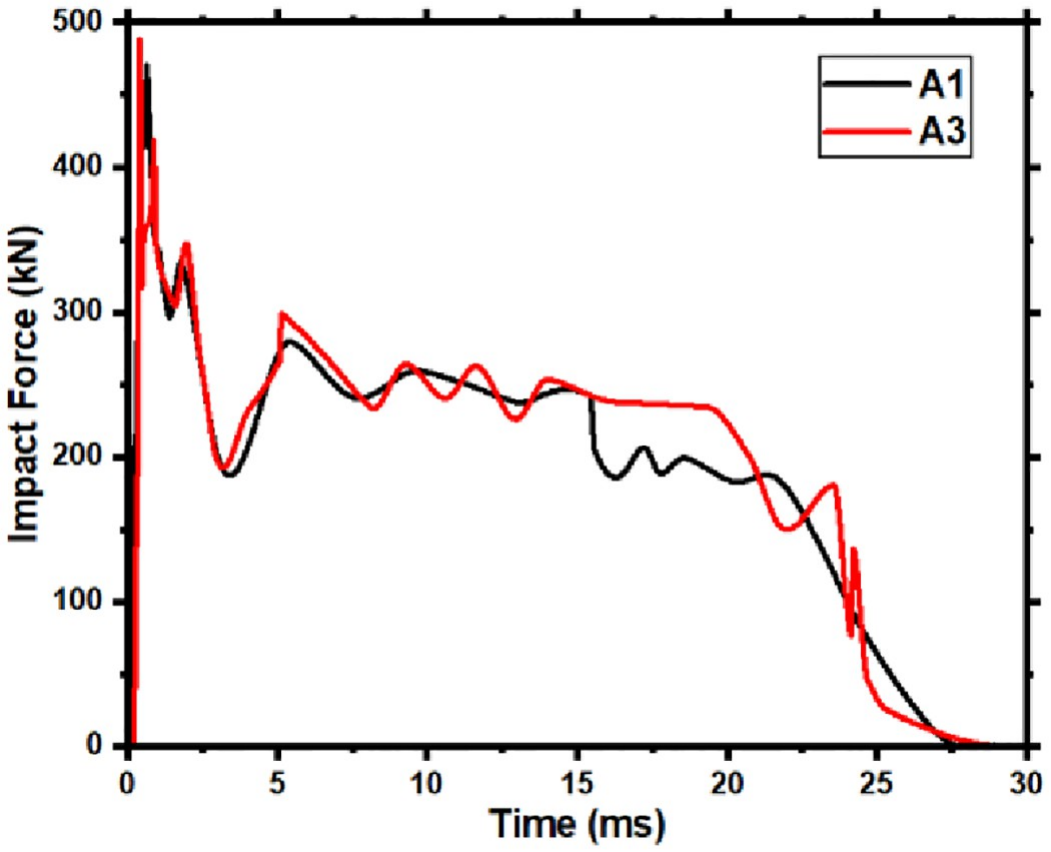
A1, A3 impact force time-history curves.

**Fig 23:**
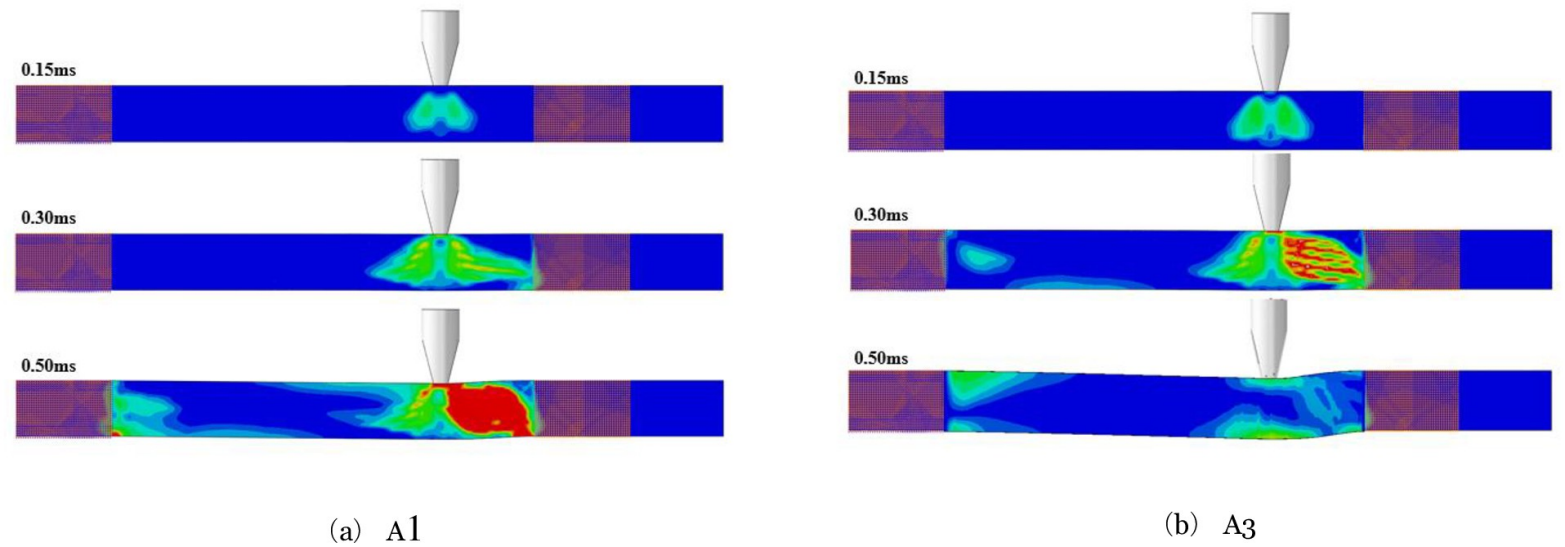
Development of stress distribution in peak stage. (a) A1, (b) A3.

**Fig 24:**
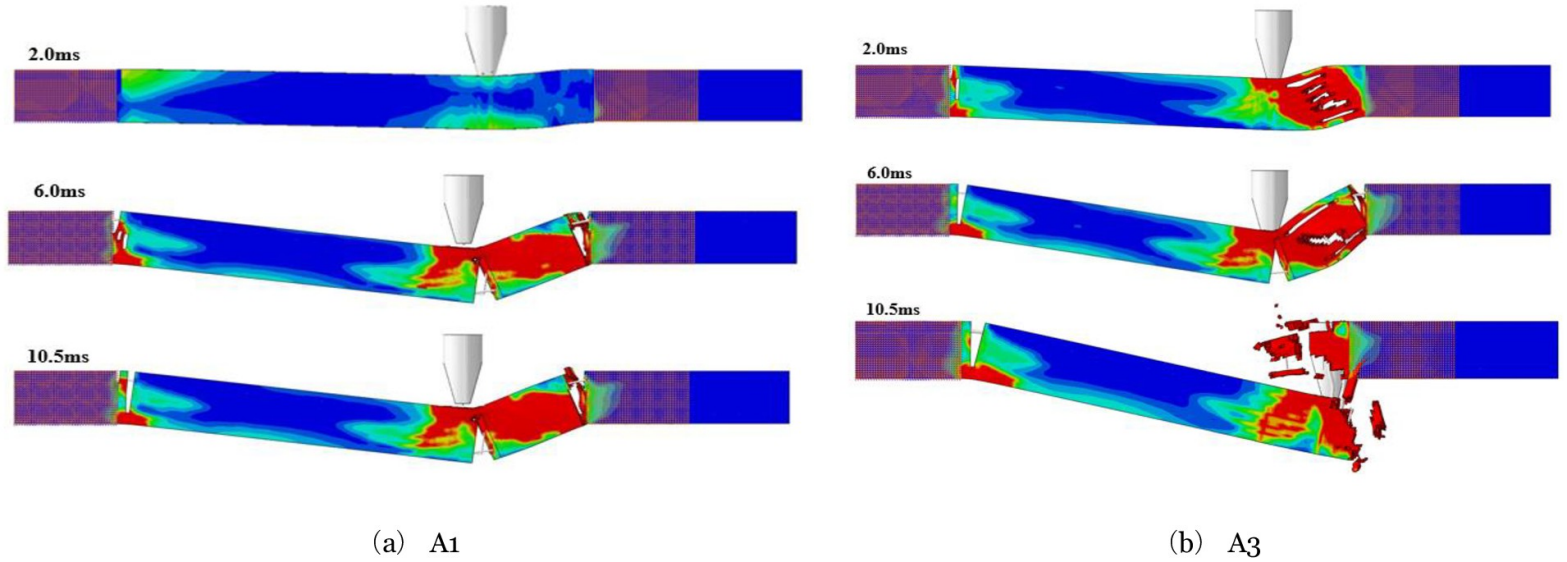
Development of stress distribution in the plateau stage. (a) A1, (b) A3.

Fig 22 shows (A1) and (A3) with factors (Four layers of CFRP thickness, impact height 5m, axial compression ratio 0, 0.3) components impact force time-history curve. The Von-Mises stress depicts in Figs 23 and 24 at the critical moment of the peak and plateau stages. By comparing the stress graph at the same moment between the two stages, analyze the influence of the axial force on the stress development law of the CFRP components under lateral impact.

During the peak impact force, the component without axial force experiences stresses only at the point of impact at 0.20ms. The top component in contact with the impacting body exhibits the highest compressive stress, while the bottom area experiences tension. Consequently, the impact point at the center of this wave forms a stress wave on either side. Stress in the middle of the component’s cross-section is minimal, almost nonexistent, a typical bending stress distribution. At 0.30ms, the peak of the impact force, the compressive stress in the previously formed stress wave continues to increase and spreads to the left and right sides of the component. Due to the proximity of the right supports, the stress reaches them first, leading to significantly higher stress in that span compared to the left side. By 0.80ms, the stress has extended to the left-span support. In the area closer to this support, high-stress concentration areas form at the top and bottom of the component. The stress rapidly develops between the impact point and the right support, resulting in high stress throughout the area.

On the other hand, the CFRP component with an axial compression ratio of 0.3 exhibits no stress at the bottom of the component at the point of impact at 0.15ms. Only the contact between the impact body and the component exhibits a high-stress concentration area, which spreads diagonally toward the middle of the component. At 0.30ms, the high-stress concentration area at the bottom is significantly smaller than in the absence of axial compression, and the stress wave is not fully closed. Similarly, at 0.80ms, the area of high-stress concentration near the left span support is also significantly smaller with axial compression.

During the impact force plateau stage, the component undergoes smooth bending deformation, with high-stress concentration areas forming at the supports on both sides and the impact position. As a result, plastic hinges form, which experience greater stresses at both the right support and the left impact. Comparing the two components reveals that when the axial compression ratio is 0, the lower part on the left side of the impact position experiences higher stress development levels and a larger development area than the upper part. However, when the axial compression ratio increases to 0.3, the upper part exhibits higher stress development levels and a larger area than the lower part. Additionally, the stress at the three plastic hinges increases more rapidly. It was observed that at 10.5ms, the bottom of the component at the impact position of A1 had developed a crack, which reduced stress levels on the left and right sides. In contrast, the A3 component experiences slower stress development and does not crack until the end of the plateau stage.

It can be seen from the development of the stress of the two components in the whole impact process mentioned above that the presence of an appropriate axial force can reduce the stress development speed of the components, especially the stress in the tensile area. Therefore, the appropriate axial force can effectively improve the impact resistance for the CFRP component with bending deformation under the asymmetrical lateral impact.

## 7. Conclusion

This paper subjected square and circular reinforced concrete columns to asymmetrical lateral impact tests. Using ABAQUS software to simulate the impact process of members under different axial compression ratios, the failure modes, maximum principal stress of concrete, shear crack penetration time, and strengthening of CFRP layers to RC members (Effect of CFRP layers thickness, impact position, bending moment, energy absorbed, and the stress of components). The following conclusions are obtained from the data analysis of impact force and deflection time history as follow:

1. It is observed through the test that when the asymmetrical-span impact occurs, the failure mode of the member is a flexural-shear failure. In square cross-section members, the greater the impact energy, the more severe the component damage; if both the impact energy and the axial force are large, the component will be severely damaged.
2. ABAQUS simulates the impact process more accurately than experimental results, with a small difference in time-history curves for impact force and deflection, suggesting that parameter selection was correct.
3. The axial force affects the dynamic response of the members under the asymmetrical lateral impact. The axial force influences the impact force’s peak value, the impact force plateau value, and the deflection curves. Through numerical simulation, it is obtained for the FH2 test member. When the axial compression ratio is in the range of 0.05~0.13, the axial force increases the impact force’s peak value and plateau value, reduces the component’s maximum deflection, and improves the impact resistance of the members. The axial force reduces the peak impact force value, increases maximum deflection, and reduces impact resistance when the axial compression ratio exceeds 0.13.
4. The failure mechanism of different axial forces affecting the impact resistance of members is obtained by analyzing the numerical simulation results. The axial force changes the maximum principal stress size and oblique cracks’ penetration speed. The axial compression ratio is in the range of 0.05~0.13, the maximum principal stress increase at the impact point of the component is small, and the penetration speed of the oblique cracks is slow, reducing the deflection of the component. Nevertheless, if the axial compression ratio exceeds 0.13, the maximum principal stress at the impact point of the member increases, local concrete damage is caused, and the penetration speed of oblique cracks is quick, which increases the deflection of components.
5. Increases in impact height increase the component’s peak impact force. Still, a small change in the plateau value of the impact force occurs, the impact duration at the plateau stage is longer, and the accumulative damage to the component increases with the thickness of the CFRP layers. There is an increase in impact force, a decrease in deformation, and an increase in impact resistance when the component is impact tested. As long as the axial force does not go beyond a certain range, the component’s impact resistance will increase, but if it goes beyond that range, it will lose its impact resistance. RC members can least resist lateral impact at the mid-span, and the impact force farther from the mid-span, the stronger the impact resistance.
6. The dynamic plastic limit bending moment at the impact position of the CFRP-RC section will increase as the axial compression ratio increases. However, the dynamic plastic limit bending moment will decrease beyond a certain axial force value. The presence of an appropriate axial force level will improve the plastic development ability of the component as a whole, resulting in an increase in the dynamic plastic limit bending moment of each member section and an increase in the duration of plastic development. Upon lateral impact, the energy consumption of steel bars in CFRP-RC components will initially decrease with increasing axial compression ratio but will eventually increase. Concurrently, the energy consumption of concrete will increase with the axial compression ratio. The presence of axial force can reduce the stress levels of CFRP-RC components under the asymmetrical lateral impact, particularly the stress in the tensile area, thereby improving the component’s flexural failure capacity.
